# Simplified algorithm for genetic subtyping in diffuse large B-cell lymphoma

**DOI:** 10.1038/s41392-023-01358-y

**Published:** 2023-04-10

**Authors:** Rong Shen, Di Fu, Lei Dong, Mu-Chen Zhang, Qing Shi, Zi-Yang Shi, Shu Cheng, Li Wang, Peng-Peng Xu, Wei-Li Zhao

**Affiliations:** 1 Shanghai Institute of Hematology, State Key Laboratory of Medical Genomics; National Research Center for Translational Medicine at Shanghai, Ruijin Hospital Affiliated to Shanghai Jiao Tong University School of Medicine, Shanghai, China; 2 Department of Pathology, Ruijin Hospital Affiliated to Shanghai Jiao Tong University School of Medicine, Shanghai, China; 3 Pôle de Recherches Sino-Français en Science du Vivant et Génomique, Laboratory of Molecular Pathology, Shanghai, China

**Keywords:** Haematological cancer, Cancer genetics, Tumour heterogeneity, Cancer microenvironment

## Abstract

Genetic classification helps to disclose molecular heterogeneity and therapeutic implications in diffuse large B-cell lymphoma (DLBCL). Using whole exome/genome sequencing, RNA-sequencing, and fluorescence in situ hybridization in 337 newly diagnosed DLBCL patients, we established a simplified 38-gene algorithm (termed ‘LymphPlex’) based on the information on mutations of 35 genes and rearrangements of three genes (*BCL2*, *BCL6*, and *MYC*), identifying seven distinct genetic subtypes: *TP53*^Mut^ (*TP53* mutations), MCD-like (*MYD88*, *CD79B*, *PIM1*, *MPEG1*, *BTG1*, *TBL1XR1*, *PRDM1*, *IRF4* mutations), BN2-like (*BCL6* fusion, *NOTCH2*, *CD70*, *DTX1*, *BTG2*, *TNFAIP3*, *CCND3* mutations), N1-like (*NOTCH1* mutations), EZB-like (*BCL2* fusion, *EZH2*, *TNFRSF14*, *KMT2D*, *B2M*, *FAS*, *CREBBP*, *ARID1A*, *EP300*, *CIITA*, *STAT6*, *GNA13* mutations, with or without *MYC* rearrangement), and ST2-like (*SGK1*, *TET2*, *SOCS1*, *DDX3X*, *ZFP36L1*, *DUSP2*, *STAT3*, *IRF8* mutations). Extended validation of 1001 DLBCL patients revealed clinical relevance and biological signature of each genetic subtype. *TP53*^Mut^ subtype showed poor prognosis, characterized by p53 signaling dysregulation, immune deficiency, and PI3K activation. MCD-like subtype was associated with poor prognosis, activated B-cell (ABC) origin, BCL2/MYC double-expression, and NF-κB activation. BN2-like subtype showed favorable outcome within ABC-DLBCL and featured with NF-κB activation. N1-like and EZB-like subtypes were predominated by ABC-DLBCL and germinal center B-cell (GCB)-DLBCL, respectively. EZB-like-MYC^+^ subtype was characterized by an immunosuppressive tumor microenvironment, while EZB-like-MYC^-^ subtype by NOTCH activation. ST2-like subtype showed favorable outcome within GCB-DLBCL and featured with stromal-1 modulation. Genetic subtype-guided targeted agents achieved encouraging clinical response when combined with immunochemotherapy. Collectively, LymphPlex provided high efficacy and feasibility, representing a step forward to the mechanism-based targeted therapy in DLBCL.

## Introduction

Diffuse large B-cell lymphoma (DLBCL), the most common subtype of non-Hodgkin’s lymphoma, represents a molecularly heterogeneous entity.^1–3^ DLBCL is potentially curable with immunochemotherapy using rituximab, cyclophosphamide, doxorubicin, vincristine, and prednisone (R-CHOP), but recurrent or progressive disease occurs in approximately 40% of the patients.^4,5^ Attempts to improve the treatment outcome by combining standard immunochemotherapy with promising novel agents targeting specific pathways have encountered difficulties likely due to the biological complexity of DLBCL.^6,7^ It is thus highly desirable to further reveal molecular heterogeneity of DLBCL by simplified algorithm, that may efficiently be translated for therapeutic use, leading to improved accuracy of outcome prediction and efficacy of the targeted therapy.

As for the classification of DLBCL, a strategy to categorize DLBCL is based upon the detection of rearrangements of *MYC*, *BCL2* and/or *BCL6* by fluorescent in-situ hybridization (FISH).^2^ The most recent revision of the WHO classification of lymphoid neoplasms recognizes the high-grade B-cell double-/triple-hit lymphoma, with *MYC* rearrangement and *BCL2* and/or *BCL6* rearrangements, as a particular entity associated with aggressive behavior and inferior outcome.^8^ Gene expression profiling allows to distinguish three molecular subtypes based on cell of origin (COO), dividing DLBCL cases into activated B-cell like (ABC), germinal center B-cell like (GCB), and unclassified subtypes.^9,10^ The COO distinction has been proved of prognostic value, as patients with ABC-DLBCL generally show less favorable responses to standard therapy than those with GCB-DLBCL.^11^ Also, the COO categorization helps to understand the heterogeneous responses of patients with DLBCL to targeted therapies such as ibrutinib, a novel therapeutic agent targeting B-cell receptor (BCR) signaling pathway.^12^ Since the COO distinction does not solely account for the divergent treatment outcomes of patients with DLBCL following either R-CHOP or targeted therapies, additional genetic complexity remains to be defined.

Genomic studies can illustrate the genetic alterations of DLBCL and facilitate the categorization of genetic subtypes.^13–17^ The GenClass algorithm first identifies four distinct genetic subtypes, known as MCD (based on co-occurrence of *MYD88*^L265P^ and *CD79B* mutations), BN2 (based on *BCL6* fusions and *NOTCH2* mutations), N1 (based on *NOTCH1* mutations), and EZB (based on *EZH2* mutations and *BCL2* fusions).^15^ Recently, the LymphGen algorithm has identified additional subtypes, including EZB-MYC (EZB subtype with or without *MYC* rearrangements), ST2 (based on *SGK1* and *TET2* mutations), and A53 (based on *TP53* mutations and deletions).^17^ Meanwhile, five molecular clusters related to COO are defined: two ABC-DLBCL groups (one with low risk and possible marginal zone origin (C1) and the other a high-risk group (C5) enriched in cases with mutations in *MYD88* and *CD79B*), two GCB-DLBCL groups (one with a favorable (C4) and the other with a poor (C3) outcome), and an ABC/GCB-independent group (C2) with biallelic inactivation of *TP53* and copy number changes.^13^ Furthermore, using clustering techniques on targeted sequencing data allows characterization of resulting clusters according to the genetic features most enriched in each cluster, for example, identification and characterization of NOTCH2-, MYD88-, BCL2-, TET2/SGK1-, and SOCS1/SGK1-assoicated clusters.^14^ Interestingly, the above genetic subtypes share partially overlapping classification systems. The MCD subtype (comparable with C5 or MYD88-associated subtype) is associated with ABC-DLBCL, contributing to hyperactivation of NF-κB with poor prognosis.^13–15,17^ The BN2 subtype (comparable with C1 or NOTCH2-associated subtype) includes mutations targeting the BCR-dependent NF-κB pathway.^13–15,17^ The EZB subtype (comparable with C3 or BCL2-associated subtype) is related to GCB-DLBCL, exhibiting frequent mutations in chromatin modifiers with favorable outcome.^13–15,17^ These findings suggest that genetic subtyping helps reveal the diversity of DLBCL in oncogenic pathway engagement, gene expression profile, prognostic value, and therapeutic vulnerabilities.

Despite the progress made, challenges remain in clinical practice regarding genetic subtyping. The high demand of specimen quality for sequencing, the relatively long duration awaiting whole exome sequencing (WES)/whole genome sequencing (WGS) bioinformatics analysis, and the complexity of clustering algorithms limited its broad application.^13–15,17^ To improve the feasibility in clinical use and permit the timely use of targeted agents based on genetic subtypes, there is a great demand to develop simplified but efficient methods for genetic subtyping. Previously, we developed a 20-gene algorithm using the information on mutations of 18 genes and rearrangements of two genes (*BCL2* and *BCL6*) based on the GenClass algorithm,^15^ and conducted a prospective, phase 2, randomized trial, to explore genetic subtype-guided targeted agents plus R-CHOP (R-CHOP-X) immunochemotherapy in newly diagnosed DLBCL patients (Guidance-1, NCT04025593).^18^ Here we further developed a 38-gene algorithm (termed ‘LymphPlex’) using information on mutations of 35 genes and rearrangements of three genes (*BCL2*, *BCL6*, and *MYC*) to identify seven genetic subtypes based on the LymphGen algorithm,^17^ including the *TP53* mutated (*TP53*^Mut^), MCD-like, BN2-like, N1-like, EZB-MYC^+^-like, EZB-MYC^-^-like, and ST2-like. We also explore the clinicopathological, prognostic, molecular, and microenvironmental attributes of these genetic subtypes in a large cohort of 1001 patients with newly diagnosed DLBCL. The LymphPlex algorithm may provide a useful tool for genetic subtyping, thereby facilitating the optimization of treatment strategies for DLBCL in the era of precision medicine.

## Results

### Simplified LymphPlex algorithm established for genetic subtyping in DLBCL

Patient baseline characteristics were summarized in Supplementary Table 1. Among the Ruijin cohort of 1001 patients, 543 (54.2%) were male, 424 (42.4%) were >60 yr, 104 (10.4%) had poor performance status, 467 (46.7%) had elevated serum lactate dehydrogenase (LDH), 457 (45.7%) had advanced Ann Arbor stage, and 266 (26.6%) had multiple extranodal involvement. Accordingly, 516 (51.5%) were at low-risk and 485 (48.5%) were at intermediate- or high-risk, as defined by International Prognostic Index (IPI). Two hundred and forty-three (25.6%) were BCL2/MYC double-expressor lymphoma. As for COO classification, 148 (31.2%) were GCB-DLBCL, 235 (49.5%) were ABC-DLBCL, and 92 (19.4%) were unclassified subtype. Comparing with the training cohort, the validation cohorts^14,19^ had increased prevalence of elderly patients and poor performance status. Patients with intermediate/high- or high-risk IPI and GCB-DLBCL were also increased.

**Fig. 1:**
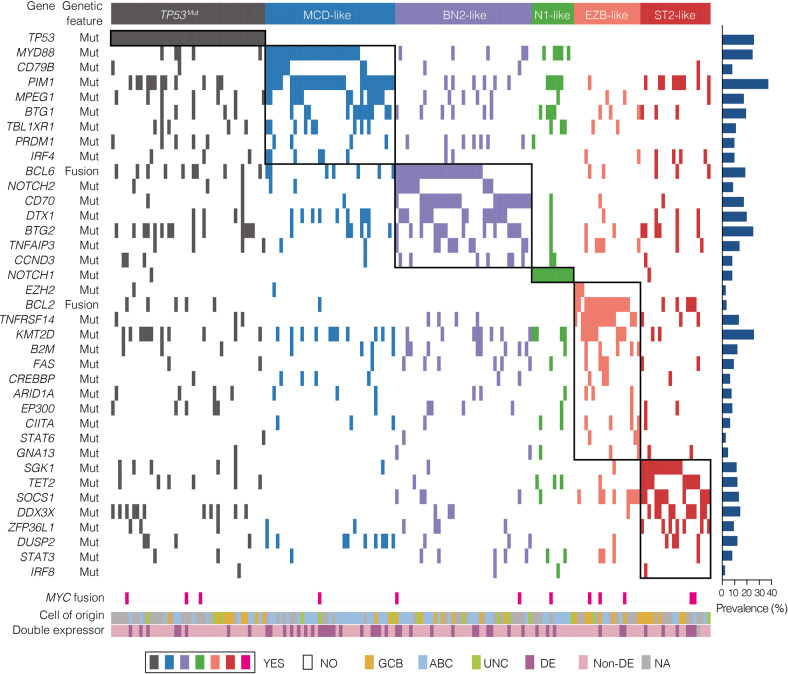
Genetic subtypes based on the LymphPlex algorithm derived from the Ruijin cohort with WES/WGS data (*n* = 337). Overall landscape of genetic subtypes based on the LymphPlex algorithm derived from the Ruijin cohort with WES/WGS and FISH data (*n* = 337). Shown on the left panel are genetic features in DLBCL samples that have been classified into each subtype. Shown on the right panel are the mutation rates of corresponding genes. Shown at bottom are *MYC* rearrangement, COO classification including GCB-DLBCL, ABC-DLBCL, and unclassified subtype, as well as BCL2/MYC double-expression (DE) status. GCB germinal center B-cell, ABC activated B-cell, UNC Unclassified

We first applied the LymphPlex algorithm based on the LymphGen algorithm^17^ in the training cohort with WES or WGS data (*n* = 337) to determine the genetic subtype classifier. The following genetic subtypes were identified: mutations in *TP53* for *TP53*^Mut^; mutations in *MYD88*, *CD79B*, *PIM1*, *MPEG1*, *BTG1*, *TBL1XR1*, *PRDM1*, and *IRF4* for MCD-like; fusion of *BCL6* (*FBCL6*) and mutations in *NOTCH2*, *CD70*, *DTX1*, *BTG2*, *TNFAIP3*, and *CCND3* for BN2-like; mutations in *NOTCH1* for N1-like; fusion of *BCL2* (*FBCL2*) and mutations in *EZH2*, *TNFRSF14*, *KMT2D*, *B2M*, *FAS*, *CREBBP*, *ARID1A*, *EP300*, *CIITA*, *STAT6*, and *GNA13* for EZB-like (with or without *MYC* fusion); and mutations in *SGK1*, *TET2*, *SOCS1*, *DDX3X*, *ZFP36L1*, *DUSP2*, *STAT3*, and *IRF8* for ST2-like subtype (Fig. 1). Among 337 patients with WES/WGS data, 171 patients (50.7%) were categorized into genetic subtypes, with the remaining 166 patients (49.3%) classified as not otherwise specified (NOS). Of the 171 classified cases, 44 cases (25.7%) were referred as *TP53*^Mut^, 37 cases (21.6%) as MCD-like, 39 cases (22.8%) as BN2-like, 12 cases (7.0%) as N1-like, 19 cases (11.1%) as EZB-like, and 20 cases (11.7%) as ST2-like subtype. Within the EZB-like subtype, three (15.8%) had *MYC* fusion. In terms of our previous 20-gene algorithm based on the GenClass algorithm,^15^ five genetic subtypes were identified: mutations in *TP53* for the *TP53*^Mut^; mutations in *MYD88*, *CD79B*, *PIM1*, *MPEG1*, *BTG1*, and *TBL1XR1* for MCD-like; *FBCL6* and mutations in *NOTCH2*, *TNFAIP3*, *CD70*, and *DTX1* for BN2-like; mutations in *NOTCH1* for N1-like; and *FBCL2* and mutations in *EZH2*, *TNFRSF14*, *CREBBP*, *EP300*, *MTOR*, and *STAT6* for EZB-like (Supplementary Fig. 1a). The newly developed LymphPlex was in principle consistent with our previous algorithm.^18^ The 17 previously unclassified patients were assigned to specific subtype by using the LymphPlex algorithm: six were assigned to BN2-like with mutations in *BTG2* and *CCND3*, one was assigned to EZB-like with mutations in *KMT2D*, *B2M*, *FAS*, *ARID1A*, *CIITA*, and *GNA13*, and 10 were assigned to the newly identified ST2-like subtype (Supplementary Fig. 1b).

### Evaluation of the LymphPlex algorithm

**Fig. 2:**
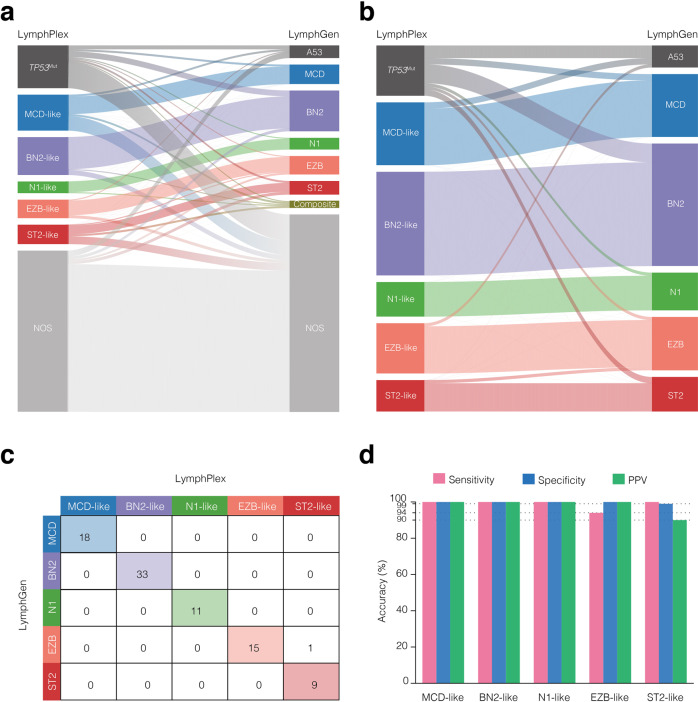
Comparison of the LymphPlex and LymphGen algorithms. **a** Sankey plots showing comparison of the LymphPlex and the LymphGen algorithms in the 337 patients with WES/WGS data including copy number data in the Ruijin cohort or **b** in the 106 cases for whom a unique genetic subtype was assigned by both algorithms. **c** Confusion matrixes showing numbers of cases for each subtype assigned by LymphPlex and LymphGen. **d** Sensitivity, specificity, and precision (positive predictive value (PPV)) of the LymphPlex algorithm for the subtype assignments, as compared to the assignments using the LymphGen algorithm

We applied both the LymphPlex and the LymphGen^17^ algorithm on the 337 patients with WES/WGS data in the Ruijin cohort. All WES/WGS mutations and copy number variants were used as the input for LymphGen. The LymphGen results were utilized as performance benchmarking. The LymphPlex algorithm assigned a genetic subtype in 50.7% (171/337) cases, while the LymphGen algorithm assigned a genetic subtype in 35.6% (120/337) cases (Fig. 2a). One hundred and six cases were classified into a unique genetic subtype by both algorithms (Fig. 2b). Excluding the *TP53*^Mut^ cases from LymphPlex and the A53 cases from LymphGen, we calculated the sensitivity, specificity, and precision (positive predictive value) of the LymphPlex algorithm for the subtype assignments in the remaining 87 cases (Fig. 2c). The LymphPlex algorithm performed acceptably, with sensitivity all above 94%, specificity all above 99%, and precision all above 90% (Fig. 2d). The LymphPlex subtypes generally belonged to the corresponding LymphGen subtypes.

### Genetic and phenotypic attributes of DLBCL genetic subtypes

**Fig. 3:**
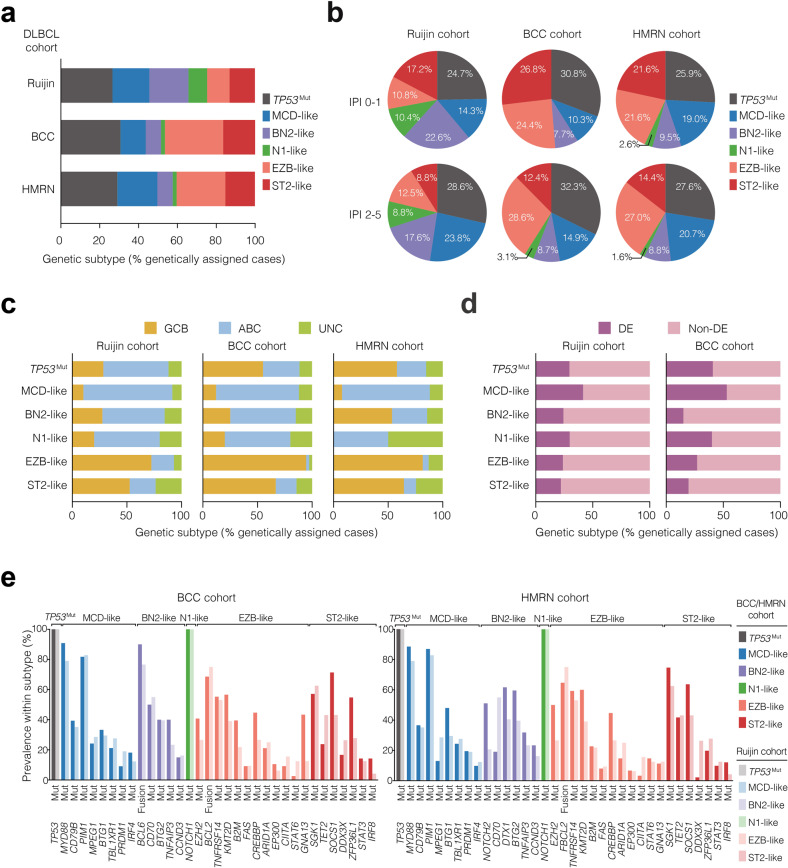
Genetic and phenotypic attributes of DLBCL genetic subtypes. **a** Distribution of genetic subtypes based on the LymphPlex algorithm. **b** Distribution of genetic subtypes according to International Prognostic Index (IPI). **c** Prevalence of COO subgroups among genetic subtypes. GCB germinal center B-cell, ABC activated B-cell, UNC Unclassified. **d** Prevalence of BCL2/MYC expression (DE) status among genetic subtypes. **e** Prevalence of the indicated genetic features among genetic subtypes defined in the BCC and HMRN cohorts, as compared to the Ruijin cohort

To evaluate the reproducibility of the LymphPlex algorithm, we analyzed the genetic and phenotypic features of the LymphPlex-assigned subtypes in the Ruijin cohort (*n* = 1001), BCC cohort (*n* = 320), and HMRN cohort (*n* = 928). The distributions of the LymphPlex-assigned subtypes were compared among three cohorts. Comparing with BCC cohort and HMRN cohort, increased incidence of the BN2-like and N1-like subtypes, but decreased incidence of the EZB-like subtype, was observed in Ruijin cohort (Fig. 3a and Supplementary Table 2). Of note, similar distribution pattern of genetic subtypes was observed in both low-risk (IPI 0–1) and intermediate- and high-risk (IPI 2–5) patients (Fig. 3b), indicating that different incidence of genetic subtypes was not resulted from uneven IPI risk group distribution among three cohorts. The clinical characteristics associated with each genetic subtype in the Ruijin cohort were also summarized in Supplementary Table 3. MCD-like had increased, while N1-like had decreased number of elderly patients. *TP53*^Mut^ and MCD-like had increased, while ST2-like had decreased prevalence of elevated serum LDH. MCD-like had increased, while ST2-like had decreased prevalence of multiple extranodal involvement. Accordingly, patients with intermediate/high- or high- risk IPI were also increased in MCD-like, while decreased in ST2-like.

Regarding COO classification, the majority of MCD-like patients were ABC-DLBCL, whereas most EZB-like patients were GCB-DLBCL (Fig. 3c), consistent with relatively lower incidence of GCB-DLBCL in Chinese patients.^14,19^ BCL2/MYC double-expression was frequently observed in the MCD-like subtype (Fig. 3d). The compositions of COO and BCL2/MYC double-expression (Fig. 3c, d) within the genetic subtypes were comparable among three cohorts, with gene mutations and *BCL2*/*BCL6* rearrangements as well (Fig. 3e). To evaluate this genetic coherence, we computed subtype-associated scores using the particular gene sets and compared the scores between gene sets, in which the subtype-defining genetic features were present or absent. In this analysis, we observed significant similarity in the genetic coherence of features defining the MCD-like, BN2-like, EZB-like, and ST2-like subtypes in the training cohort and two validation cohorts (*P* ≤ 1.3 × 10^–9^, Supplementary Table 4). The *TP53*^Mut^ and N1-like subtypes could not be evaluated by this method since *TP53*^Mut^ and N1-like subtypes were dominated by *TP53* mutations and *NOTCH1* mutations, respectively.

### Prognostic value of DLBCL genetic subtypes

**Fig. 4:**
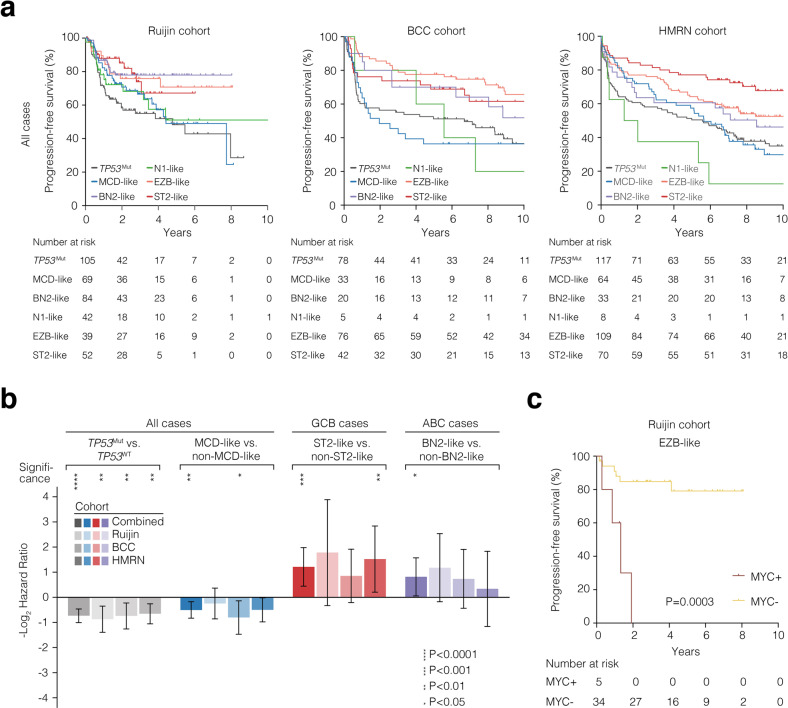
Prognostic attributes of DLBCL genetic subtypes. **a** Kaplan–Meier plots for PFS in all cases of the indicated DLBCL cohorts. **b** Hazard ratios (-log2 transformed) for the comparisons between genetic subtypes in the indicated DLBCL cohorts. **c** Kaplan–Meier plots for PFS in the EZB-like cases of the Ruijin cohort

We next examined the prognostic impact of the LymphPlex-assigned genetic subtypes in each cohort. Survival analysis was performed on the patients received R-CHOP. The *TP53*^Mut^ and MCD-like subtypes presented relatively inferior progression-free survival (PFS), as compared to the BN2-like, EZB-like, and ST2-like subtypes (Fig. 4a). Among GCB-DLBCL, the ST2-like subtype was relatively favorable; among ABC-DLBCL, the BN2-like subtype was relatively favorable (Supplementary Fig. 2). Given these consistent trends in survival, we integrated data from all three cohorts to estimate combined hazard ratios (Fig. 4b). In this model, PFS of the *TP53*^Mut^ subtype was inferior to the *TP53*^WT^ patients (*P* < 0.0001); PFS of the MCD-like subtype was inferior to all non-MCD-like subtypes (*P* = 0.0038); PFS of the ST2-like subtype was superior to all non-ST2-like subtypes within GCB-DLBCL (*P* = 0.0005); PFS of the BN2-like subtype was superior to all non-BN2-like subtypes within ABC-DLBCL (*P* = 0.0233). In the Ruijin cohort, we sub-divided the EZB-like subtype according to *MYC* rearrangement. Within the EZB-like subtype, PFS of the MYC^+^ cases were significantly inferior to that of the MYC^-^ cases (*P* = 0.0003, Fig. 4c). The MYC^+^ subset presented relatively higher IPI risk and more BCL2/MYC double-expression cases (Supplementary Fig. 3a, b). All cases in the MYC^+^ subset were GCB-DLBCL, while only 66.7% cases in the MYC^-^ subset were GCB-DLBCL (25.0% were ABC-DLBCL and 8.3% were unclassified subtype, Supplementary Fig. 3c). The gene alterations were similar in the MYC^+^ and MYC^-^ subsets of the EZB-like subtype (Supplementary Fig. 3d). As for the overall survival (OS), upon R-CHOP treatment, *TP53*^Mut^ patients showed unfavorable outcome in the Ruijin cohort (*P* = 0.0153), BCC cohort (*P* = 0.0042), and HMRN cohort (*P* = 0.0007), as compared to *TP53*^WT^ patients (Supplementary Fig. 4a). Furthermore, the OS showed significant differences among genetic subtypes assigned by the LymphPlex algorithm in the BCC cohort and HMRN cohort (*P* = 0.0005 and *P* < 0.0001, respectively), but not in the Ruijin cohort, probably because 27.0% of the patients had a follow-up less than 5 years (Supplementary Fig. 4b).

Given the distinct prognostic value of the *TP53*^Mut^ subtype, we further investigated the genetic signature of the *TP53*^Mut^ subtype. Among the 147 *TP53*^Mut^ patients in the Ruijin cohort, 116 patients (78.9%) possessed mutations in the DNA binding domain (DBD), 16 patients (10.9%) possessed mutations in the non-DBD, and 15 patients (10.2%) possessed multiple *TP53* mutations (Supplementary Fig. 5a). Survival analysis was performed on the patients received R-CHOP. No differences on PFS were observed in the patients with DBD and non-DBD *TP53* mutations (Supplementary Fig. 5b). To determine the clinical and biological impact of *TP53* mutations on DLBCL, we analyzed the molecular alterations accompanied with *TP53* mutations (Supplementary Fig. 5c). *TP53* mutations were associated with increased frequency of *KMT2D* mutations in all three cohorts. Additionally, in the Ruijin cohort, *TP53*^Mut^ patients had significantly increased mutations in *EP300* and *NOTCH2*, but decreased mutations in *SOCS1* and *CD70*. In the BCC cohort, *TP53*^Mut^ patients had significantly decreased *APF36L1* mutations. In the HMRN cohort, *TP53*^Mut^ patients had significantly increased mutations in *EP300* and *NOTCH1*, but decreased mutations in *MYD88*, *EZH2*, and *IRF4*. With respect to gene functions, in the Ruijin cohort, *TP53*^Mut^ patients presented decreased mutations associated with response to interferon-γ. In the HMRN cohort, *TP53*^Mut^ patients had decreased mutations associated with T cell activation, but increased mutations associated with Histone/DNA methylation (Supplementary Fig. 5d and Supplementary Table 5).

### Gene expression pattern of DLBCL genetic subtypes

**Fig. 5:**
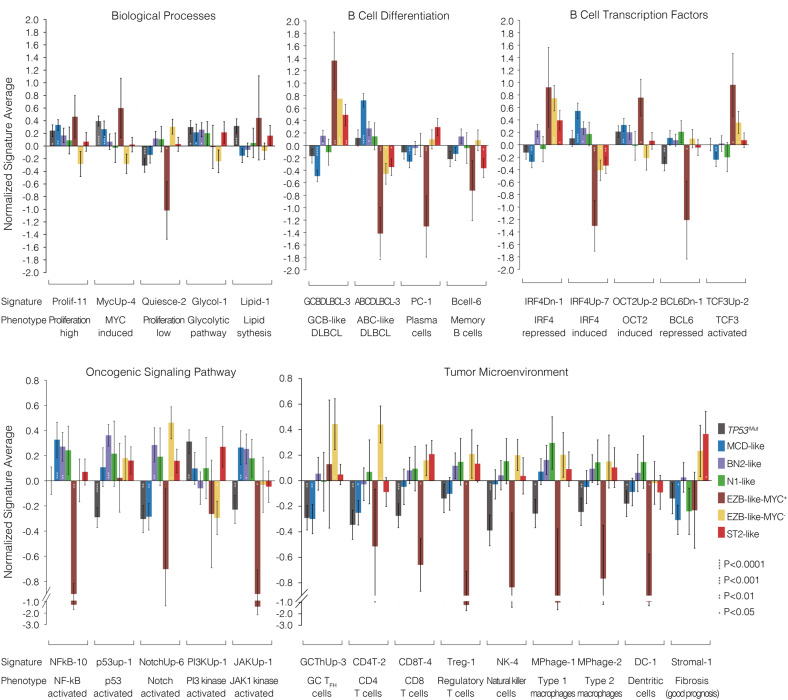
Gene expression signature according to DLBCL genetic subtypes. Shown is average normalized expression of signature genes in each subtype versus other DLBCL samples in the Ruijin cohort (*n* = 475)

To better understand the biological signatures of genetic subtypes, we analyzed gene expression profile using RNA sequencing (RNA-seq) data of 475 patients in the Ruijin cohort (Fig. 5). The subtypes differed with respect to various biological processes, with the *TP53*^Mut^ and MCD-like subtypes highly expressing signatures of cell proliferation and MYC oncoprotein, with the *TP53*^Mut^ and EZB-like-MYC^+^ subtypes expressing low level signatures of quiescence. Metabolic distinctions among genetic subtypes included highly expressing signatures of glycolytic pathway in the *TP53*^Mut^, MCD-like, and BN2-like subtypes, and high expressing signatures of lipid synthesis in the *TP53*^Mut^ subtype.

The subtypes also appeared to vary from different B cell stage. The MCD-like subtype revealed a predominance of ABC-DLBCL, while the EZB-like and ST2-like subtypes expressed signatures of GCB-DLBCL. The BN2-like subtype was consisted of both GCB- and ABC-DLBCL. The MCD-like and ST2-like subtypes had low expression of plasma cell signatures and memory B cell signatures, respectively. Among transcriptional factors, genes induced by IRF4 were overexpressed in the MCD-like and BN2-like subtypes, genes induced by OCT2 were highly expressed in the MCD-like and EZB-like-MYC^+^ subtypes, genes repressed by BCL6 were lowest in the *TP53*^Mut^ and EZB-like-MYC^+^ subtypes, and genes transactivated by TCF3 were highest in the EZB-like subtype. With respect to oncogenic pathways, NF-κB signaling was activated in the MCD-like and BN2-like subtypes, but not in the EZB-like-MYC^+^ subtype. The expression of p53-target genes was lowest in the *TP53*^Mut^ subtype. NOTCH signaling was activated in the BN2-like and EZB-like-MYC^-^ subtypes. PI3K signaling was activated in the *TP53*^Mut^ subtype. JAK2 signaling was activated in the MCD-like and BN2-like subtypes.

The tumor microenvironment alterations of genetic subtypes were also strikingly discordant: the *TP53*^Mut^ subtype generally presented low expression of all immune signatures, the MCD-like subtype presented low expression of T cell signatures, the EZB-like-MYC^+^ subtype had low expressing CD8^+^T cell signatures, while the EZB-like-MYC^–^ subtype had high expressing GC T_FH_ and CD4^+^T cell signatures. The stromal-1 signatures, which are prognostically favorable and reflect a fibrotic, macrophage-rich microenvironment, were upregulated in the ST2-like subtype, consistent with their relatively favorable outcomes.

To investigate the potential effect of the LymphPlex algorithm on mechanism-based targeted therapy in DLBCL, we applied R-CHOP in combination with different targeted agents based on the LymphPlex algorithm (R-CHOP-X). Indeed, the *TP53*^Mut^ patients were treated with intravenous decitabine, the MCD-like, BN2-like, and N1-like patients were treated with BTK inhibitors (ibrutinib, zanubrutinib, or orelabrutinib), the EZB-like, ST2-like, and NOS patients were treated with lenalidomide (Supplementary Fig. 6a). Forty-eight patients were available for response assessment, including 13 *TP53*^Mut^, 12 MCD-like, 10 BN2-like, one N1-like, one EZB-like, one ST2-like, and 10 NOS patients (Supplementary Table 6). The overall and complete response rates were 91.7% and 83.3%, respectively (Supplementary Fig. 6b). In terms of genetic subtypes, the overall response rates of the *TP53*^Mut^, MCD-like, BN2-like, N1-like, EZB-like, ST2-like, and NOS patients were 92.3% (12/13), 91.7% (11/12), 100% (10/10), 100% (1/1), 100% (1/1), 100% (1/1), and 80.0% (8/10), respectively; the complete response rates of the *TP53*^Mut^, MCD-like, BN2-like, N1-like, EZB-like, ST2-like, and NOS patients were 76.9% (10/13), 83.3% (10/12), 90.0% (9/10), 100% (1/1), 100% (1/1), 100% (1/1), and 80.0% (8/10), respectively (Supplementary Fig. 6c).

## Discussion

In the present study, we successfully developed a simplified LymphPlex algorithm and defined distinct genetic subtypes, termed as *TP53*^Mut^, MCD-like, BN2-like, N1-like, EZB-like-MYC^+^, EZB-like-MYC^-^, and ST2-like. The clustering of the LymphPlex algorithm showed a good concordance with that of the LymphGen algorithm. Integrating transcriptomic profiling with these genetic subtypes, we provided further evidence of molecular features and suggested potential targeted approaches in DLBCL.

*TP53* mutations are critically involved in tumor progression and indicate poor prognosis in DLBCL.^20–22^ Despite diversity in gene mutations correlated with *TP53* mutations among the studied cohorts, we showed similar alterations of response to interferon-γ and T cell activation, indicative of an essential role of *TP53* mutations on immune evasion.^23^ Accordingly, gene expression pattern confirmed dysregulation of p53 signaling and deficiency of anti-tumor immunity. The *TP53*^Mut^ subtype generally presented low expression of immune signatures including T cells, NK cells, macrophages, and dendritic cells. As reported in our recent phase 1/2 study, decitabine improved clinical efficacy of R-CHOP on *TP53*^Mut^ DLBCL through targeting tumor immune microenvironment.^23,24^ Given the above evidence that *TP53* mutations had significant biological features and specific response to decitabine treatment, we grouped patients with *TP53* mutations as a specific entity of DLBCL, different from previous subtyping as A53 and C2.^13,17^ Meanwhile, PI3K signaling and lipid synthesis were altered, suggesting that PI3K inhibitors and lipid metabolism reprogramming may also be effective options for the *TP53*^Mut^-subtype DLBCL.^25,26^

The MCD-like subtype was characterized by the co-occurrence of *MYD88* and *CD79B* mutations.^13,14,17^ We found that the MCD-like subtype was associated with ABC-DLBCL, BCL2/MYC double-expression, and poor prognosis. Subsequent investigation using gene expression data showed strong enrichment in ABC-DLBCL signatures, NF-κB activation, as well as IRF4 and MYC upregulation.^17,27^ BTK inhibitors targeting BCR and NF-κB signaling are promising agents for the MCD-like subtype.^12,28^ Recent report from the PHOENIX trial further proved that addition of ibrutinib improves the survival of young DLBCL patients with the MCD subtype.^29^ The BN2-like subtype was featured by *FBCL6* and *NOTCH2* mutations and comprised of ABC-, GCB- and unclassified DLBCL.^13,14,17^ In our cohort, the BN2-like subtype presented favorable outcome among ABC-DLBCL patients, consistent with the results from the NCI study.^17^ Gene expression profiling also revealed the signatures of BCR-dependent NF-κB activation, indicating the potential response to BTK inhibitors.^17^ The N1-like subtype was dominated by *NOTCH1* mutations, mainly consisted of ABC-DLBCL, and exhibited poor prognosis.^14^ Limited biological feature and therapeutic targets were disclosed, however, ibrutinib was reported to bring survival benefit in N1 subtype according to the PHOENIX trial.^29^

The EZB-like subtype was characterized by *FBCL2* and *EZH2* mutations.^13,14,17^ This group revealed a predominance of GCB-DLBCL and was generally associated with favorable outcome. According to *MYC* rearrangement, the EZB-like-MYC^+^ subtype was distinguished by poor prognosis and CD8^+^T cell deficiency within tumor microenvironment. Lenalidomide showed survival benefit in ABC-DLBCL and may also had an impact on immunomodulation in the EZB-like-MYC^+^ subtype.^7,30,31^ In the EZB-like-MYC^-^ DLBCL, NOTCH pathway was activated, suggesting possible effect of NOTCH inhibitors. In addition, EZH2 inhibitors may be effective in treating the EZB-like DLBCL patients bearing *EZH2* mutations via suppressing methyltransferase activity.^32^ The ST2-like subtype was distinguished by mutations in *SGK1*, *TET2*, and *SOCS1*.^13,14,17^ Predominantly GCB-DLBCL in origin, the ST2-like subtype was associated with favorable outcome. With respect to gene expression profile, the expression of stromal signatures was increased in the ST2-like subtype, which could be related to stroma modulation induced by *SOCS1* mutations and resulted in attenuated tumor growth.^33^ Moreover, *TET2* mutations were reported to be involved in T-cell deficiency. These results may indicate potential efficacy of lenalidomide in ST2-like DLBCL through modulating tumor immune microenvironment.^34^ To try to translate LymphPlex into clinical practice, we applied R-CHOP in combination with different targeted agents selected by LymphPlex to treat 48 patients with newly diagnosed DLBCL and showed encouraging results. A prospective, phase 3, randomized trial of genetic subtype-guided R-CHOP-X immunochemotherapy in newly diagnosed DLBCL (Guidance-02, NCT05351346) is ongoing.

In conclusion, the simplified LymphPlex algorithm of genetic subtyping displayed high efficacy and clinical practicability in DLBCL. LymphPlex is implemented with a small number of gene alterations revealed by targeted sequencing and FISH and can be applied to paraffin biopsies instead of frozen biopsies, which reveals a good availability for clinical use. Also, the LymphPlex results can be output using targeted sequencing data instead of WES/WGS data, which simplifies the process of data analysis and ensure its application in multicenter clinical trials and timely use of targeted agents based on genetic subtypes. This simple but efficient algorithm facilitated to disclose molecular heterogeneity in DLBCL, thereby contributing to the design of the mechanism-based therapy in the era of precision medicine.

## Materials and methods

### Patients and procedures

**Fig. 6:**
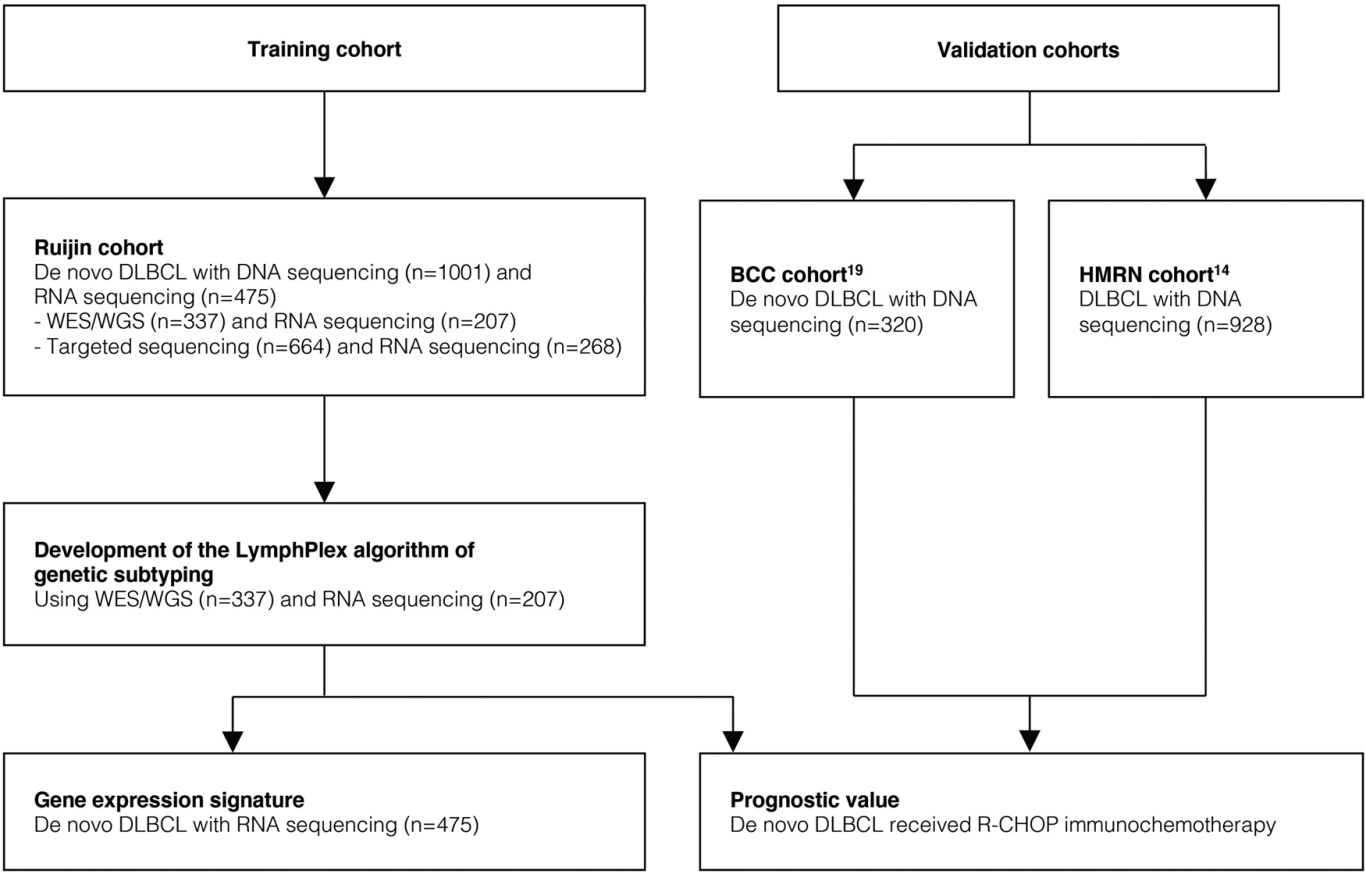
Flow chart of the study. DLBCL diffuse large B-cell lymphoma, WES whole-exome sequencing, WGS whole-genome sequencing, R-CHOP rituximab, cyclophosphamide, doxorubicin, vincristine, and prednisone

This study was conducted on 1001 patients with newly diagnosed DLBCL (Fig. 6). The patients were included from May 2006 to December 2020, with the last follow-up through September 2021. Histological diagnosis was established according to World Health Organization (WHO) Classification.^35^ WES and WGS were performed on 228 and 109 patients with available tumor samples, respectively. The remaining 664 cases were analyzed with targeted sequencing data covering 35 lymphoma-associated genes (*ARID1A*, *B2M*, *BTG1*, *BTG2, CCND3, CD70*, *CD79B*, *CIITA*, *CREBBP*, *DDX3X*, *DTX1*, *DUSP2, EP300*, *EZH2*, *FAS, GNA13, IRF4, IRF8, KMT2D, MPEG1*, *MYD88*, *NOTCH1*, *NOTCH2*, *PIM1*, *PRDM1*, *SGK1*, *SOCS1*, *STAT3*, *STAT6*, *TBL1XR1*, *TET2*, *TNFAIP3*, *TNFRSF14*, *TP53*, and *ZFP36L1*), as well as rearrangements in *BCL2*, *BCL6*, and *MYC*. A total of 475 patients were available for RNA-seq, including 207 cases of WES/WGS group and 268 cases of targeted sequencing group.

Clinical parameters included gender, age, Eastern Cooperative Oncology Group performance status, Ann Arbor stage, serum LDH, and number of extranodal involvement. As for BCL2/MYC double-expressor lymphoma, cut-off value of BCL2 and MYC were 50% and 40%, respectively.^35^ COO classification including GCB-DLBCL, ABC-DLBCL, and unclassified subtype was determined using RNA-seq.^36^ Rearrangements of *BCL2*, *BCL6*, and *MYC* were assessed by FISH. Survival analysis was performed on 730 patients received R-CHOP immunochemotherapy, excluding those received immunochemotherapy other than R-CHOP (*n* = 227) or chemo-free regimen (*n* = 44). The study was approved by the Shanghai Ruijin Hospital Review Board, and informed consent was obtained in accordance with the Declaration of Helsinki.

### Sample processing

Genomic DNA (gDNA) was extracted from frozen tumor tissues using a QIAamp DNA Mini Kit (Qiagen, Hilden, Germany), or from formalin-fixed paraffin-embedded (FFPE) tumor tissues using a GeneRead DNA FFPE Tissue Kit (Qiagen, Hilden, Germany), based on the manufacturer’s guidelines. The concentration of gDNA was measured using Qubit 3.0 Fluorometer (Thermo Fisher Scientific, Waltham, MA, USA), and the gDNA was quantified by Qsep100 System (BIOptic, China).

Total RNA was extracted from frozen tumor tissues using Trizol and RNeasy Mini Kit (Qiagen, Hilden, Germany). RNA quantity was assessed on Nanodrop, and the integrity of total RNA was estimated by RNA 6000 Nano Kit on Aligent 2100 Bioanalyzer.

### Whole-exome sequencing (WES)/whole-genome sequencing (WGS)

WES was performed on the frozen tumor tissues of 146 patients and the FFPE tumor tissues of 82 patients. Whole exome capture was performed using the SeqCap EZ Human Exome kit (version 3·0), and sequencing was performed on HiSeq 4000 platform with 150 bp paired-end strategy (Shanghai Rightongene Biotechnology Co., Ltd, Shanghai, China). WGS was performed on the frozen tumor tissues of 109 patients. Detailly, library was validated by Agilent 2100 Bioanalyzer, and sequencing was performed on Illumina HiSeq platform with 150 bp paired-end strategy in WuXi NextCODE (Shanghai, China). Also, 42 randomly selected matched peripheral blood samples were submitted to WES (*n* = 25, divided into five groups) and WGS (*n* = 17) to build a somatic mutation calling principle, thereafter, excluding the germ-line polymorphisms. The quality control data of 315 samples received WES/WGS were described as previously reported.^37^ The median depth of the additional samples measured with WES was 158.0X (IQR, 147.1X, 179.6X), with a median mapping ratio of 99.5% (IQR, 98.2%, 99.5%), a median Q30 of 90.6% (IQR, 90.0%, 90.9), and a median 100X coverage rate of 86.2% (IQR, 85.5%, 91.4%).

After sequencing, read pairs were aligned to Human Reference Genome version hg19 (downloaded from UCSC Genome Browser, URLs) by Burrows-Wheeler Aligner^38^ (BWA) (version 0·7·13-r1126). Samtools^39^ (version 1.3) was used to generate chromosomal coordinate-sorted bam files and to remove PCR duplications. The reads were then realigned around potential indel regions by Genome Analysis Toolkit^40^ (GATK) (version 3·4) IndelRealigner with the recommended pipeline. GATK Haplotype Caller and GATK Unified Genotyper were applied to call SNVs (single nucleotide variations) and Indels (Insertion and deletion). Mutation detection and analysis of BAM files were performed using the cancer genome analysis program Mutect2 at the Broad Institute and annotated with ANNOVAR. Noticeably, to reduce systematic error for FFPE samples as the formaldehyde deaminates cytosines and thereby results in C → T transition mutations, the F1R2 and F2R1 annotations were adopted and FilterByOrientationBias was performed to filter the orientation bias.

Next, all the somatic functional mutations, including nonsynonymous SNVs, frameshift or in-frame indels, stopgain or stoploss were obtained. Visual inspection was used to exclude potential false positive results. Homemade pipeline was used to filter SNVs and indels detected by the above software, based on the following criteria: (1) variants with mapping quality >30 were retained; (2) SNVs or Indels with a mutation allele frequency (MAF) <0.001 in databases of 1000 genomes project, 1000 genome East Asian, ExAC all or ExAC East Asian and genomAD were retained; (3) variant allele frequency (VAF) >5% and read depth >10; (4) dbSNP (v147) sites existed COSMIC database (the Catalog of Somatic Mutations in Cancer, version 77) were retained; (5) SNPs or Indels including stopgain, stoploss, frameshift, nonframeshift and splicing sites were retained; (6) duplicate frameshifts found in multiple samples, variants found in repetitive regions, variants found in regions with poor coverage were excluded; (7) germline variants found in control samples or detected by sanger sequencing in paired peripheral blood samples were excluded. A list of variants called was provided in the Supplementary Information (see Supplementary Table 7).

Copy number analyses of WGS/WES samples were conducted with CNVkit^41^ using recommended pipeline (https://cnvkit.readthedocs.io/en/stable/pipeline.html). GISTIC 2.0^42^ (*q* < 0.1) was used to verify the.cnv files and determined the copy number profiles.

### Targeted sequencing

Targeted sequencing of the lymphoma-related genes was performed on 664 FFPE tumor samples using MultipSeq Custom Panel (Shanghai Rightongene Biotechnology Co., Ltd, China). The capture probes were designed based on ~0.39 Mb genomic regions of the lymphoma-related genes that are frequently mutated in DLBCL, other common lymphoma and hematologic malignancies. In summary, gDNA was fragmented, end repaired, linked with sequencing adapters, purified and went through pre-PCR using Enzyme Plus Library Prep Kit (iGeneTech, Beijing, China). After hybridization and concentration, sequencing was performed on the Novaseq (Illumina) sequencing platform. The mean depth of each sample measured with targeted sequencing was 1261X (IQR, 1085X, 1482X), with a median mapping ratio of 97.0% (IQR, 95.8%, 99.0%), a median Q30 of 93.6% (IQR, 92.9 %, 94.4%), and a median 200X coverage rate of 96.0% (IQR, 93.0%, 97.0%).

After sequencing, the quality of the raw sequencing data was assessed using FastQC software (version 1.11.4). In addition, Trimmomatic (version 3.6) software was used to process raw sequencing data to remove adaptor sequences and low-quality fragments. The data processing process of Broad Institute was used to process all sequence data, and the original sequencing sequence was aligned with the human reference genome hg19 using BWA (0·7·13-r1126). The repeats were eliminated, and the base quality was recalibrated. GATK was used for SNP calling. Mutation detection and analysis of BAM files were performed using the cancer genome analysis program Mutect2 at the Broad Institute and annotated with ANNOVAR. Also, F1R2 and F2R1 annotations were adopted and FilterByOrientationBias was performed to filter the orientation bias. The SNVs and Indels were screened based on the filtering conditions: (1) variants with mapping quality >30 were retained; (2) SNVs or Indels with a mutation allele frequency (MAF) <0.001 in databases of 1000 genomes project, 1000 genome East Asian, ExAC all or ExAC East Asian and genomAD were retained; (2) SNVs or Indels with a VAF ≥5% was retained; (3) dbSNP (v147) sites existed COSMIC database were retained; (4) SNPs or Indels including stopgain, stoploss, frameshift, nonframeshift and splicing sites were retained; (5) missense mutation type require meet the conditions of sift ≤ 0.05, Polyphen2_HVAR_pred ≥0.447 and CADD > 4 were retained. A list of variants called was provided in the Supplementary Information (see Supplementary Table 7).

### RNA sequencing

RNA purification, reverse transcription, library construction and sequencing were performed in WuXi NextCODE according to the manufacturer’s instructions (Illumina San Diego, CA, USA). PolyA mRNA was purified from total RNA using oligo-dT-attached magnetic beads and then fragmented by fragmentation buffer. The synthesized cDNA was subjected to end-repair, phosphorylation, and ‘A’ base addition according to Illumina’s library construction protocol. Then Illumina sequencing adapters were added to both size of the cDNA fragments. After PCR amplification for DNA enrichment, the target fragments of 200–300 bp were cleaned up. After library construction, Qubit (Thermo Fisher Scientific) was used to quantify concentration of the resulting sequencing libraries, while the size distribution was analyzed using Agilent BioAnalyzer 2100 (Agilent). After library validation, Illumina cBOT cluster generation system with HiSeq PE Cluster Kits (Illumina) was used to generate clusters. Paired-end sequencing was performed using an Illumina HiSeq system following Illumina-provided protocols for 2 × 150 paired-end sequencing.

Read pairs were aligned to Refseq hg19 by STAR (version 020201). The HTSeq was applied to generate table files containing transcript counts.^43^ Further analyses were performed by R (v4.0.2). Voom function from R package “limma” (v3.38.3) was used to remove batch effect and normalize raw reads.^44^ R package “clusterProfiler” (v3.10.1) was used for Gene Ontology enrichment analysis.^45^

### Simplified LymphPlex algorithm

LymphPlex was a novel clustering strategy, based on the LymphGen algorithm^17^ but designed in a simplified manner. More specifically, a given DLBCL sample was assigned into one of the defined genetic subtypes (TP53^Mut^, MCD-like, BN2-like, N1-like, EZB-like with or without *MYC* rearrangements, and ST2-like) by applying the Partitioning Around Medoids (PAM) method with mutation data of 35 genes and rearrangement data of three genes *BCL2*, *BCL6*, and *MYC*.^46^ These mutated genes (see Supplementary Table 8) were identified from WES and WGS of 337 patients and also were supported by RNA-seq data available for 207 patients (that is, differentially expressed and involved in the functional pathways as revealed by Gene Ontology enrichment analysis). Patients with *TP53* mutations were assigned into a particular subtype with a higher priority. The set of features considered for possible association with a class are based on those used in the LymphGen algorithm: *TP53*^Mut^ (*TP53* mutations), MCD-like (*MYD88*, *CD79B*, *PIM1*, *MPEG1*, *BTG1*, *TBL1XR1*, *PRDM1*, *IRF4* mutations), BN2-like (*BCL6* fusion, *NOTCH2*, *CD70*, *DTX1*, *BTG2*, *TNFAIP3*, *CCND3* mutations), N1-like (*NOTCH1* mutations), EZB-like (*BCL2* fusion, *EZH2*, *TNFRSF14*, *KMT2D*, *B2M*, *FAS*, *CREBBP*, *ARID1A*, *EP300*, *CIITA*, *STAT6*, *GNA13* mutations, with or without *MYC* rearrangement), and ST2-like (*SGK1*, *TET2*, *SOCS1*, *DDX3X*, *ZFP36L1*, *DUSP2*, *STAT3*, *IRF8* mutations). Computational procedures on the LymphPlex algorithm are shared on https://github.com/difuSJTU/LymphPlex. The online version of the LymphPlex algorithm is provided on https://kylinmu.shinyapps.io/LymphPlexR/.

### Validation DLBCL cohorts

To evaluate the LymphPlex, we used two validation cohorts: the BC Cancer (BCC) cohort which consisted of 320 newly diagnosed DLBCL patients received R-CHOP, with available clinical and genomic data from a targeted gene sequencing panel and FISH results,^19^ and the UK population-based Hematological Malignancy Research Network (HMRN) cohort which consisted of 928 DLBCL patients from a real-world study (617 patients received R-CHOP), with available clinical and panel-based DNA sequencing data.^14^

### Statistical analysis

Fisher’s exact test was used for between-categorical data comparisons. Differences of normalized gene expression in two groups were analyzed using Mann–Whitney *U* test. PFS was measured from the date of diagnosis to the date when disease progression/relapse was recognized or the date of last follow-up. OS was calculated from the date of diagnosis to the date of death or the date of last follow-up. Survival analysis was performed using the Kaplan–Meier curves and Cox proportional hazards regression models. Statistical significance was defined as two-sided *P* < 0.05. The above statistical analyses were performed by Statistical Package for the Social Sciences (SPSS) v26.0 (SPSS Inc., Chicago, IL) and R v4.0.2 (R foundation, Vienna, Austria).

## Supplementary information

Supplementary Materials

Supplementary Table 2

Supplementary Table 6

Supplementary Table 7

## Data Availability

Genomic and gene expression data have been deposited on https://www.biosino.org/node in project OEP001143. Proposals requesting individual participant data that underlie the results reported in this paper (after de-identification) can be sent to zhao.weili@yahoo.com.
