# Supplementary material for: Simplified algorithm for genetic subtyping in diffuse large B-cell lymphoma
Source: Signal Transduct Target Ther. 2023 Apr 10;8:145. doi: 10.1038/s41392-023-01358-y (PMC10083170; doi:10.1038/s41392-023-01358-y)
Supplement: Supplementary file 1 — Supplementary Materials [file 41392_2023_1358_MOESM1_ESM.docx]

Supplementary Materials for

Simplified algorithm for genetic subtyping in

diffuse large B-cell lymphoma

Rong Shen^1*^, Di Fu^1*^, Lei Dong^2*^, Mu-Chen Zhang^1^, Qing Shi^1^, Zi-Yang Shi^1^, Shu Cheng^1^, Li Wang^1,3^, Peng-Peng Xu^1†^, Wei-Li Zhao^1,3†^

**†** Correspondence to: Wei-Li Zhao, Email: zhao.weili@yahoo.com. Peng-peng Xu, Email: pengpeng_xu@126.com.

**This PDF file includes:**

Supplementary Figures (Figure S1-S6)

Supplementary Tables (Table S1, S3-S5, and S8)

**Other Supplementary Materials for this manuscript include the following (separate file):**

Supplementary Table 2. Clinical and genetic features of the 1001 DLBCL patients in the Ruijin cohort.

Supplementary Table 6. Clinical features of the 48 DLBCL patients treated with R-CHOP-X.

Supplementary Table 7. Mutations of the 1001 patients in the Ruijin cohort.

**Supplementary Figure 1. Association between the LymphPlex algorithm and the 20-gene algorithm
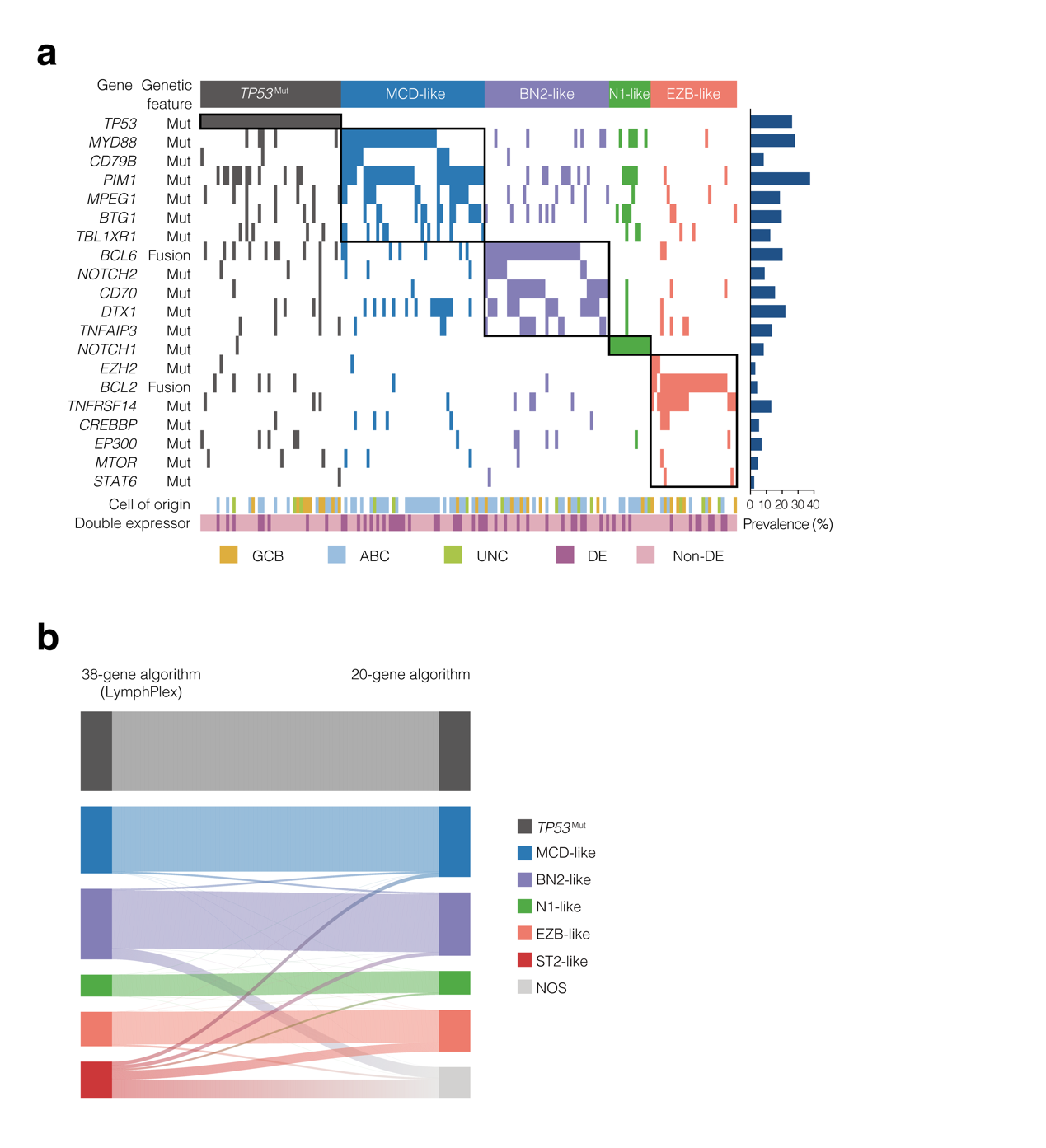
**

(a) Genetic subtypes based on the 20-gene algorithm derived from the Ruijin cohort with WES/WGS data (n = 337). GCB, germinal center B-cell; ABC, activated B-cell; UNC, Unclassified; DE, double expressor.

(b) Sankey plot showing the corresponding genetic subtype assigned by the 20-gene algorithm in the 171 cases for whom a genetic subtype was assigned by the LymphPlex algorithm.

**Supplementary Figure 2. PFS according to the genetic subtypes within GCB or ABC cases.**

**
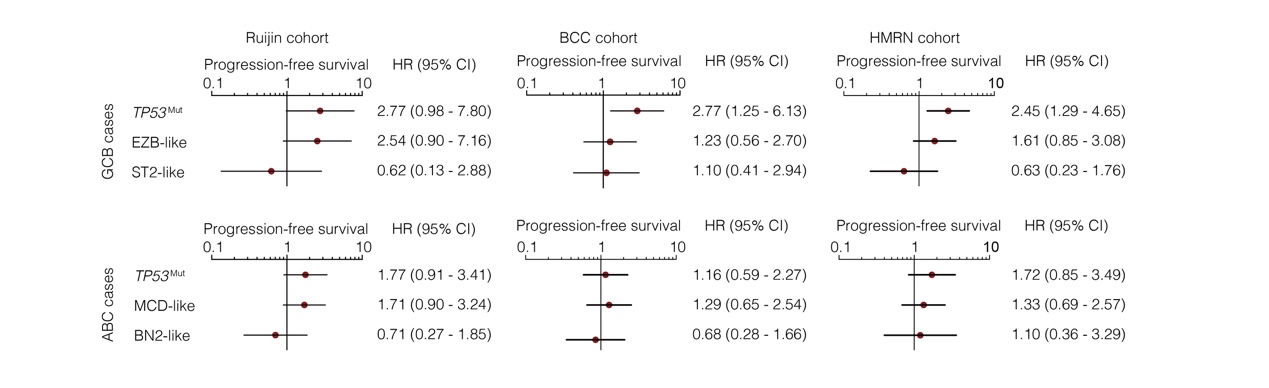
**

Forest plots of hazard ratios for PFS within GCB or ABC cases of the indicated DLBCL cohorts. Hazard ratios are shown relative to the NOS patients.

**Supplementary Figure 3. Subgroups of EZB-like subtype according to *MYC* rearrangement.
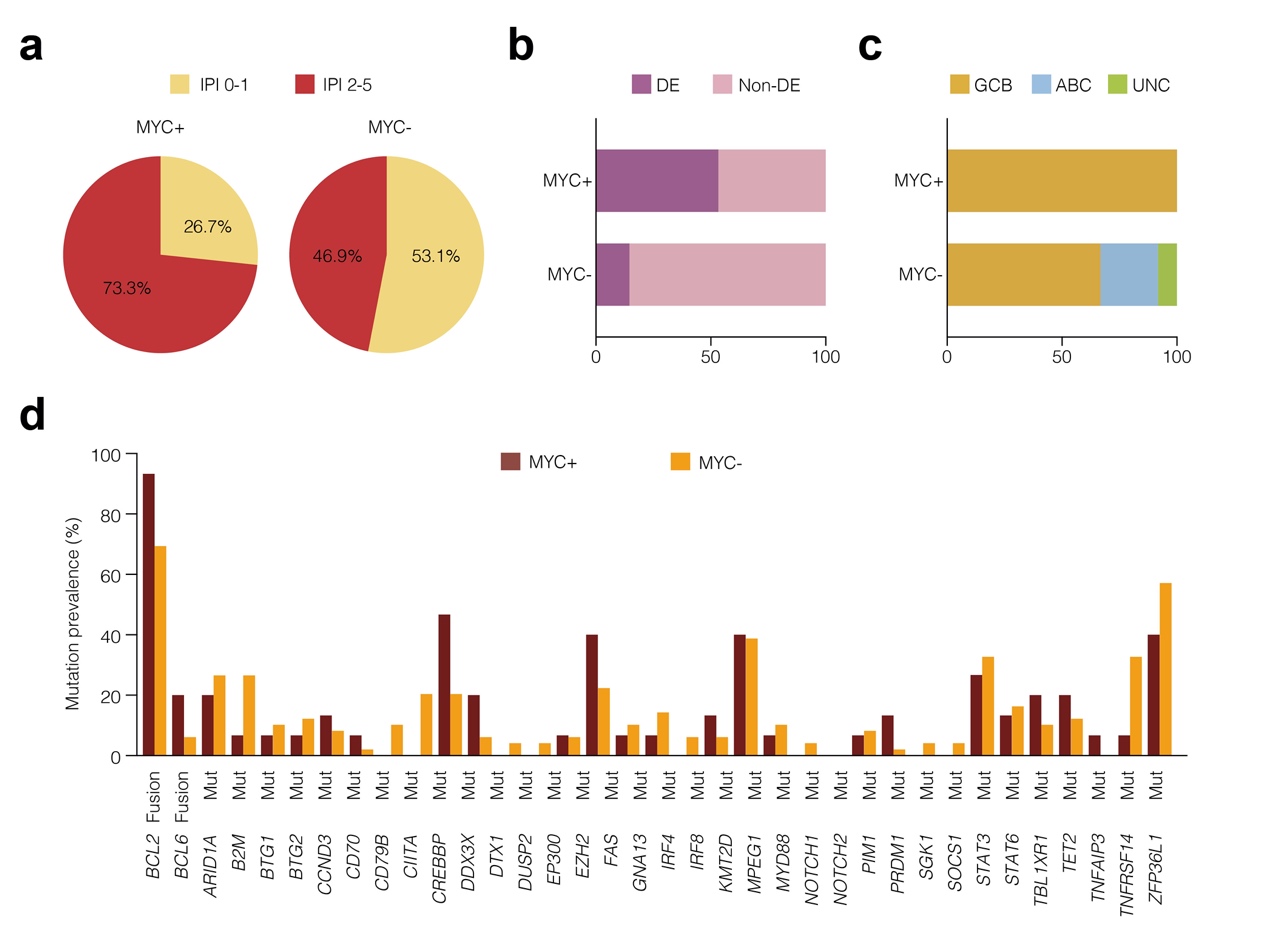
**

Comparison of (a) IPI risk, (b) BCL2/MYC double-expression, (c) cell-of-origin classification, and (d) mutation prevalence between the subgroups of EZB-like subtype according to *MYC* rearrangement in the Ruijin cohort.

**Supplementary Figure 4. Overall survival in DLBCL genetic subtypes.**

**
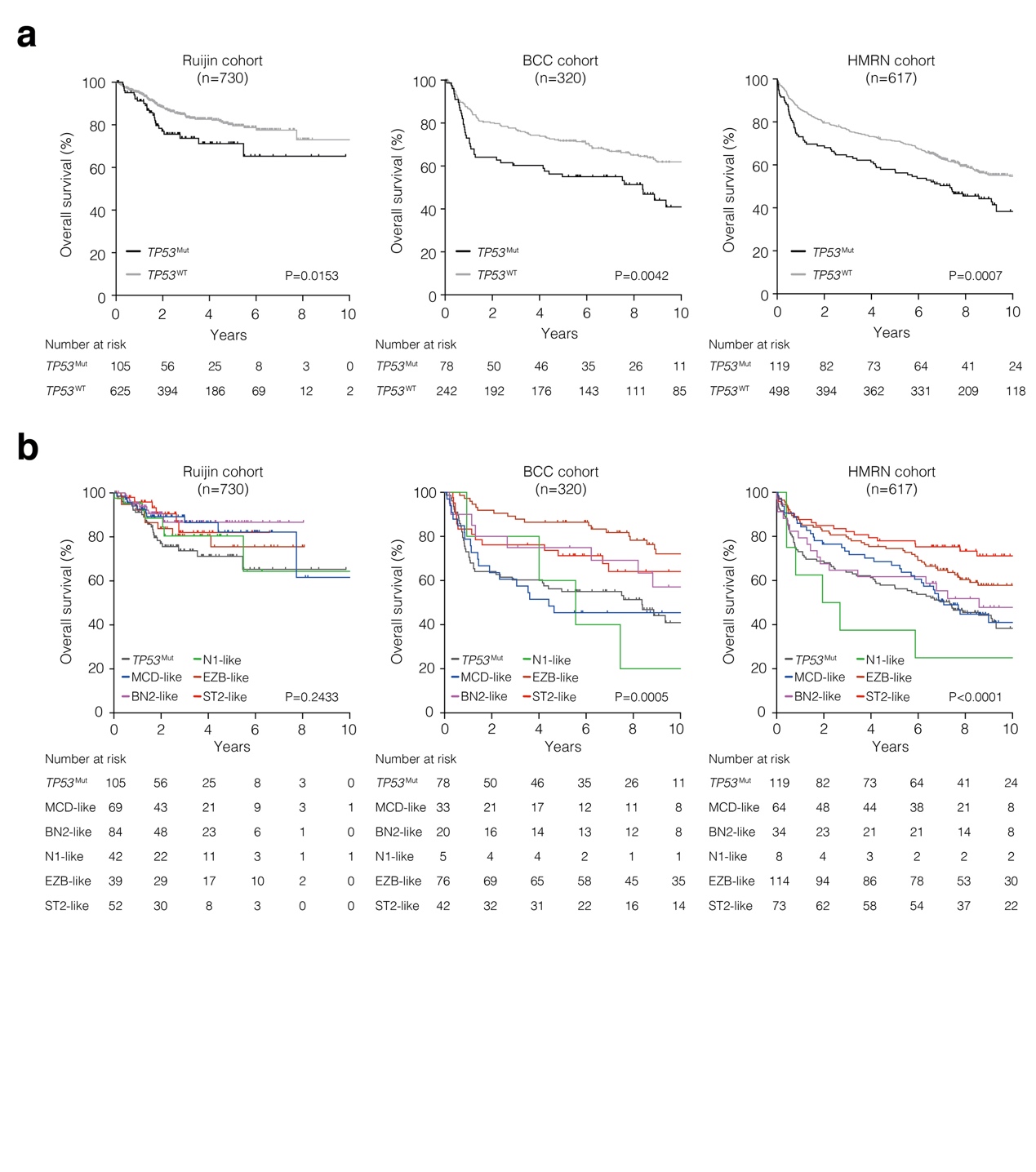
**

Kaplan-Meier plots of overall survival according to (a) mutation status of TP53 and (b) genetic subtypes in the indicated DLBCL cohorts.

**Supplementary Figure 5. Genetic signature of *TP53*^Mut^ subtype.**

**
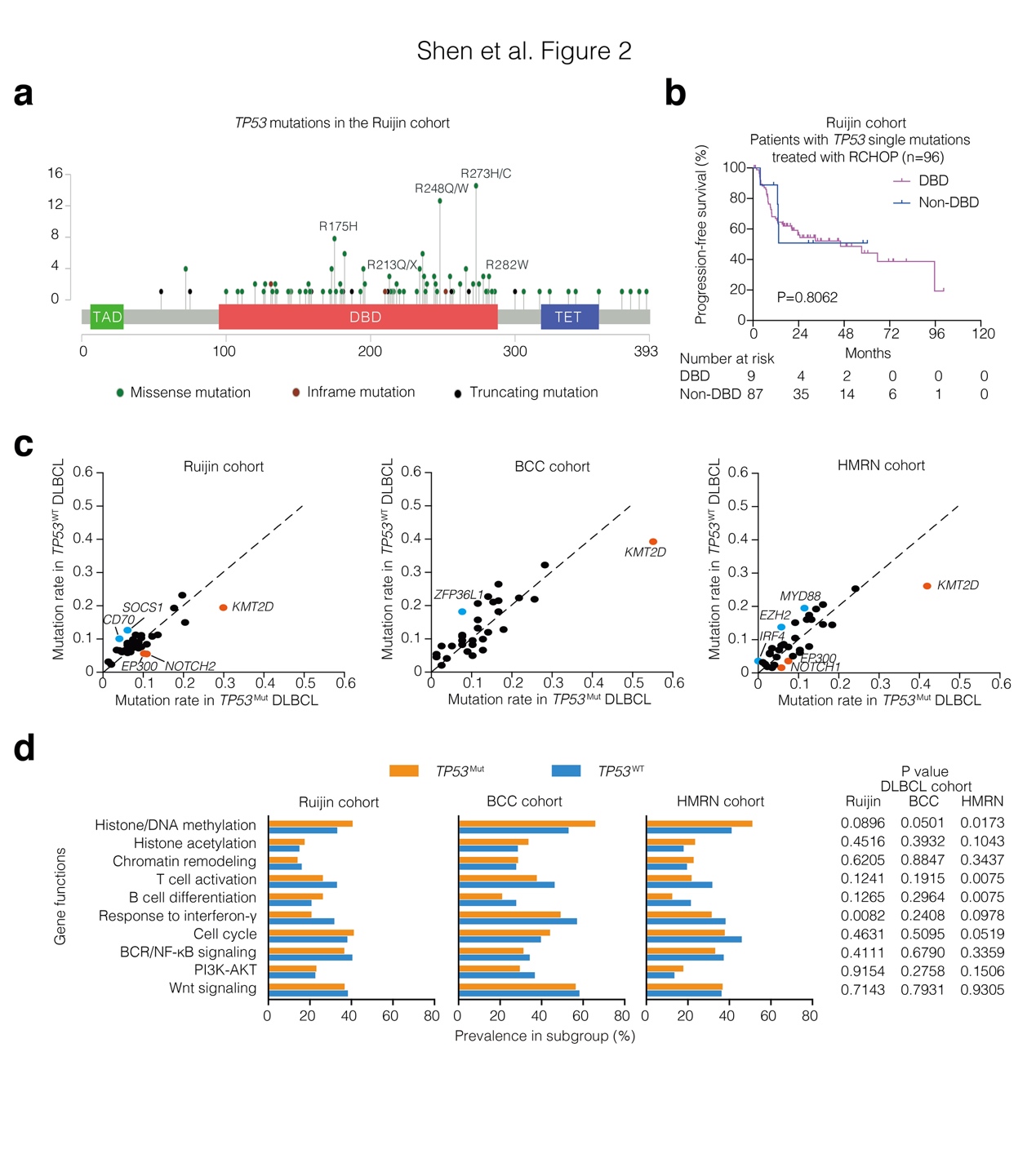
**

(a) Lollipop plot illustrating specific localization of *TP53* variants. TAD, transcription-activation domain; DBD, DNA binding domain; TET, tetramerization motif.

(b) Kaplan-Meier plot of PFS according to *TP53* mutational status in the Ruijin cohort.

(c) Distribution of recurrently mutated genes according to mutational status of *TP53.*

(d) Prevalence of mutations associated with gene functions according to mutational status of *TP53*.

**Supplementary Figure 6. R-CHOP-X in newly diagnosed DLBCL (n=48).**


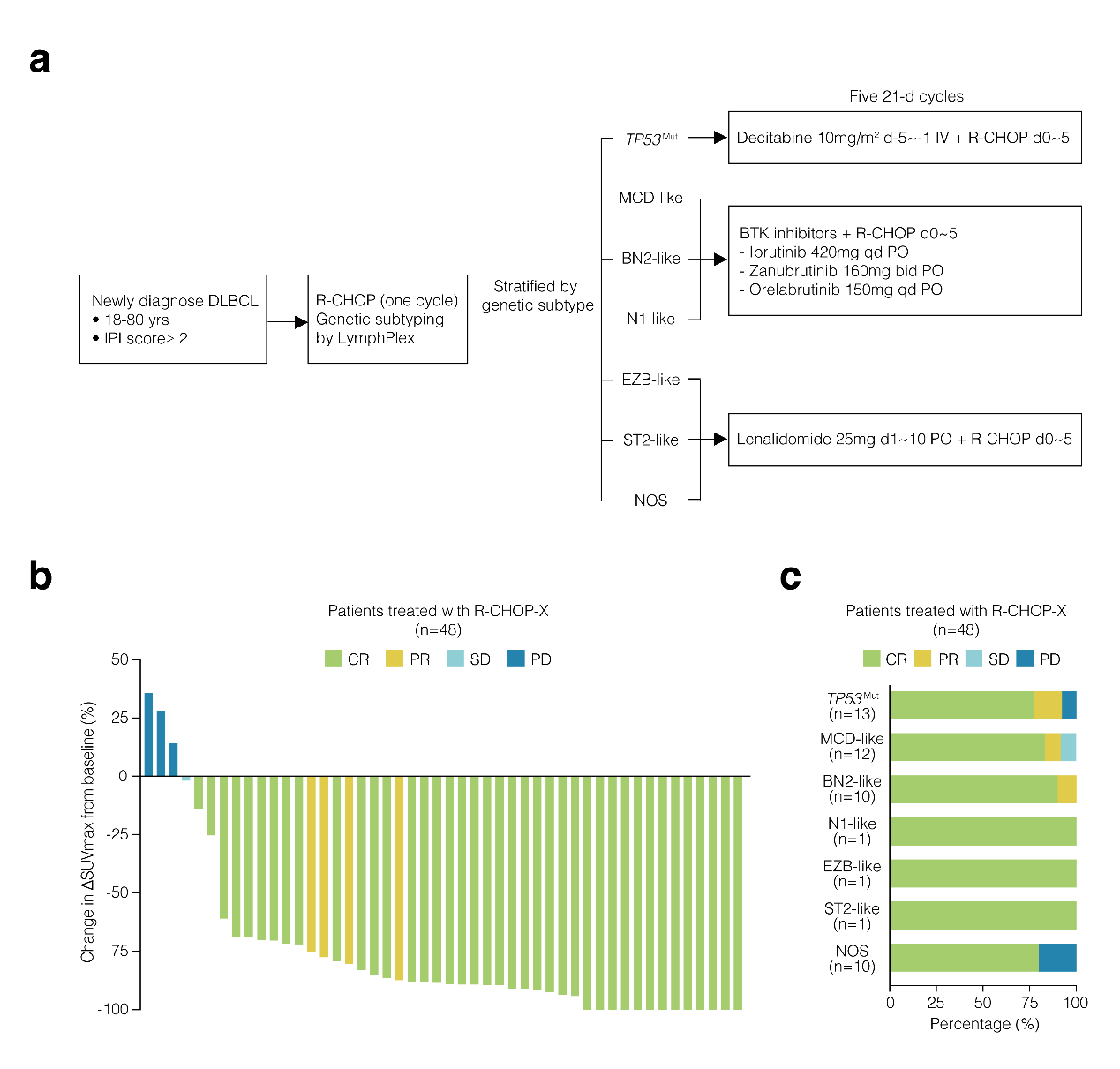


(a) Protocol of R-CHOP-X treatment.

(b) Clinical efficacy of R-CHOP-X based on LymphPlex.

(c) Subgroup analysis of clinical response according to genetic subtypes.

CR, complete response; PR, partial response; SD, stable disease; PD, progressive disease.

**Supplementary Table 1. Clinical characteristics of DLBCL patients.**

| **Cohort characteristics** | **Ruijin cohort** | **BCC cohort** | **HMRN cohort** |
| --- | --- | --- | --- |
| Number of patients | 1001 | 320 | 928 |
| Male gender | 543 (54.2) | 204 (63.8) | 500 (53.9) |
| Age, >60yr | 424 (42.4) | 189 (59.1) | 651 (70.2) |
| Performance status (ECOG), ≥2* | 104 (10.4) | 102 (32.2) | 256 (30.3) |
| Elevated LDH* | 467 (46.7) | 155 (52.9) | NA |
| Ann Arbor stage, III-IV* | 457 (45.7) | 165 (52.1) | 478 (61.0) |
| Extranodal involvement, ≥2* | 266 (26.6) | 43 (13.6) | NA |
| IPI* |  |  |  |
| 0-1 | 516 (51.5) | 104 (35.0) | 213 (31.4) |
| 2-5 | 485 (48.5) | 193 (65.0) | 466 (68.6) |
| BCL-2/MYC double expression† | 243 (25.6) | 97 (31.0) | NA |
| Cell of origin† |  |  |  |
| GCB | 148 (31.2) | 183 (57.2) | 265 (50.5) |
| ABC | 235 (49.5) | 103 (32.2) | 142 (27.0) |
| Unclassified | 92 (19.4) | 34 (10.6) | 118 (22.5) |

Data are presented as n (%) unless otherwise indicated. NA, not available; ECOG, Eastern Cooperative Oncology Group; LDH, lactate dehydrogenase; IPI, International Prognostic Index.

*Percentage of those with data available.

†Percentage of those with material available.

**Supplementary Table 3. Clinical characteristics associated with the genetic subtypes assigned by LymphPlex.**

| Characteristics | Genetic subtypes, n (%) | | | | | | | P value |
| --- | --- | --- | --- | --- | --- | --- | --- | --- |
|  | *TP53*^Mut^ | MCD-like | BN2-like | N1-like | EZB-like | ST2-like | NOS |  |
| Number of patients | 147 | 105 | 111 | 53 | 64 | 72 | 449 |  |
| Age>60yr | 53 (36.1) | 60 (57.1)^**^ | 40 (36.0) | 15 (28.3)^*^ | 23 (35.9) | 36 (50.0) | 197 (43.9) | 0.0017 |
| ECOG≥2 | 17 (11.6) | 17 (16.2) | 9 (8.1) | 2 (3.8) | 10 (15.6) | 7 (9.7) | 42 (9.4) | 0.1444 |
| Elevated LDH | 87 (59.2)^**^ | 62 (59.0)^**^ | 47 (42.3) | 22 (41.5) | 30 (46.9) | 23 (31.9)^**^ | 196 (43.7) | 0.0003 |
| Ann Arbor III-IV | 70 (47.6) | 52 (49.5) | 48 (43.2) | 28 (52.8) | 34 (53.1) | 23 (31.9)^*^ | 202 (45.0) | 0.1628 |
| ENI≥2 | 41 (27.9) | 40 (38.1)^*^ | 20 (18.0)^*^ | 12 (22.6) | 21 (32.8) | 11 (15.3)^*^ | 124 (27.6) | 0.0064 |
|  |  |  |  |  |  |  |  |  |
| IPI |  |  |  |  |  |  |  | 0.0070 |
| (0-1) | 69 (46.9) | 40 (38.1)^**^ | 63 (56.8) | 29 (54.7) | 30 (46.9) | 48 (66.7)^**^ | 237 (52.8) |  |
| (2-5) | 78 (53.1) | 65 (61.9) | 48 (43.2) | 24 (45.3) | 34 (53.1) | 24 (33.3) | 212 (47.2) |  |

ECOG, Eastern Cooperative Oncology Group; LDH, lactate dehydrogenase; ENI, extranodal involvement; IPI, International Prognostic Index.

^*^ P-value vs. others <0.05

^**^ P-value vs. others <0.01

**Supplementary Table 4. Statistical analysis of subtype-defining genetic features.**

| **Genetic subtype** | **Probability that subtype-defining genetic features co-occur** | | |
| --- | --- | --- | --- |
|  | **Ruijin cohort** | **BCC cohort** | **HMRN cohort** |
| MCD | <1.0E-16 | 1.11E-16 | <1.0E-16 |
| BN2 | 5.55E-15 | 6.20E-14 | 1.33E-09 |
| EZB | <1.0E-16 | <1.0E-16 | <1.0E-16 |
| ST2 | 7.77E-15 | 8.63E-13 | 2.22E-15 |

**Supplementary Table 5. Gene functions associated with recurrent mutated genes.**

| **Gene functions** | **Genes** |
| --- | --- |
| Chromatin organization |  |
| Histone/DNA methylation | *EZH2, KMT2D, TET2* |
| Histone acetylation | *CREBBP, EP300* |
| Chromatin remodeling | *ARID1A, SGK1* |
| Immune response |  |
| T cell activation | *CD70, MPEG1, PRDM1, TNFRSF14, ZFP36L1* |
| Response to interferon-γ | *B2M, CIITA, IRF4, IRF8, SOCS1* |
| Cell cycle | *BTG1, BTG2, CCND3, DTX1, FAS* |
| Signaling pathway |  |
| BCR/NF-κB signaling | *CD79B, DTX1, MYD88, TNFAIP3* |
| PI3K-AKT | *DDX3X, GNA13, IRF4, NOTCH1* |
| Wnt signaling | *DDX3X, GNA13, IRF4, PIM1, TBL1XR1* |

**Supplementary Table 8. Pathways influenced by indicated mutations.**

| **Gene** | **Gene Ontology terms** | **Set size** | **ES** | **NES** | **p value** | **q value** |
| --- | --- | --- | --- | --- | --- | --- |
| ARID1A | GO_CHROMATIN_ASSEMBLY_OR_DISASSEMBLY | 180 | -0.39 | -1.66 | 0.0036 | 0.0104 |
| ARID1A | GO_NOTCH_SIGNALING_PATHWAY | 189 | 0.39 | 1.50 | 0.0054 | 0.0112 |
| ARID1A | GO_RESPONSE_TO_INTERFERON_GAMMA | 195 | -0.35 | -1.53 | 0.0076 | 0.0129 |
| ARID1A | GO_CYTOKINE_PRODUCTION | 747 | -0.33 | -1.59 | 0.0086 | 0.0139 |
| B2M | GO_RESPONSE_TO_INTERFERON_GAMMA | 195 | 0.49 | 1.96 | 0.0021 | 0.0138 |
| B2M | GO_CYTOKINE_PRODUCTION | 747 | 0.33 | 1.52 | 0.0021 | 0.0138 |
| B2M | GO_REGULATION_OF_T_HELPER_1_TYPE_IMMUNE_RESPONSE | 27 | 0.52 | 1.49 | 0.0365 | 0.1233 |
| BTG1 | GO_CYTOKINE_PRODUCTION | 747 | 0.32 | 1.37 | 0.0011 | 0.0540 |
| BTG1 | GO_REGULATION_OF_INNATE_IMMUNE_RESPONSE | 433 | 0.31 | 1.30 | 0.0204 | 0.1687 |
| BTG2 | GO_CYTOKINE_MEDIATED_SIGNALING_PATHWAY | 770 | -0.24 | -1.22 | 0.0099 | 0.1365 |
| BTG2 | GO_REGULATION_OF_SIGNAL_TRANSDUCTION_BY_P53_CLASS_MEDIATOR | 175 | 0.36 | 1.41 | 0.0095 | 0.1365 |
| CCND3 | GO_REGULATION_OF_INNATE_IMMUNE_RESPONSE | 433 | 0.29 | 1.35 | 0.0028 | 0.0214 |
| CCND3 | GO_GLUCOSE_METABOLIC_PROCESS | 117 | -0.41 | -1.58 | 0.0036 | 0.0214 |
| CCND3 | GO_REGULATION_OF_SIGNAL_TRANSDUCTION_BY_P53_CLASS_MEDIATOR | 175 | 0.41 | 1.75 | 0.0024 | 0.0214 |
| CD70 | GO_CYTOKINE_PRODUCTION | 747 | 0.33 | 1.50 | 0.0021 | 0.0175 |
| CD70 | GO_REGULATION_OF_NOTCH_SIGNALING_PATHWAY | 96 | 0.39 | 1.45 | 0.0099 | 0.0541 |
| CD70 | GO_I_KAPPAB_KINASE_NF_KAPPAB_SIGNALING | 261 | 0.30 | 1.28 | 0.0188 | 0.0757 |
| CD70 | GO_GLYCOLYTIC_PROCESS | 104 | 0.35 | 1.32 | 0.0411 | 0.1215 |
| CD79B | GO_REGULATION_OF_INNATE_IMMUNE_RESPONSE | 433 | 0.28 | 1.30 | 0.0052 | 0.0493 |
| CD79B | GO_CYTOKINE_PRODUCTION | 747 | 0.26 | 1.26 | 0.0068 | 0.0596 |
| CD79B | GO_REGULATION_OF_B_CELL_RECEPTOR_SIGNALING_PATHWAY | 27 | 0.50 | 1.54 | 0.0372 | 0.1971 |
| CIITA | GO_RESPONSE_TO_INTERFERON_GAMMA | 195 | 0.47 | 1.98 | 0.0026 | 0.0147 |
| CIITA | GO_CYTOKINE_PRODUCTION | 747 | 0.31 | 1.49 | 0.0038 | 0.0147 |
| CIITA | GO_REGULATION_OF_T_HELPER_1_TYPE_IMMUNE_RESPONSE | 27 | 0.60 | 1.77 | 0.0044 | 0.0147 |
| CIITA | GO_REGULATION_OF_T_CELL_MEDIATED_IMMUNITY | 68 | 0.44 | 1.57 | 0.0089 | 0.0262 |
| CREBBP | GO_REGULATION_OF_T_CELL_MEDIATED_IMMUNITY | 68 | -0.54 | -1.97 | 0.0024 | 0.0181 |
| CREBBP | GO_CHROMATIN_ASSEMBLY_OR_DISASSEMBLY | 180 | -0.59 | -2.50 | 0.0026 | 0.0181 |
| CREBBP | GO_CYTOKINE_PRODUCTION | 747 | -0.29 | -1.42 | 0.0036 | 0.0181 |
| CREBBP | GO_RESPONSE_TO_INTERFERON_GAMMA | 195 | -0.32 | -1.38 | 0.0053 | 0.0216 |
| CREBBP | GO_REGULATION_OF_T_HELPER_1_TYPE_IMMUNE_RESPONSE | 27 | -0.54 | -1.65 | 0.0102 | 0.0354 |
| CREBBP | GO_REGULATION_OF_T_HELPER_17_TYPE_IMMUNE_RESPONSE | 19 | -0.61 | -1.70 | 0.0106 | 0.0354 |
| CREBBP | GO_METHYLATION | 356 | 0.30 | 1.30 | 0.0211 | 0.0548 |
| DDX3X | GO_COLLAGEN_FIBRIL_ORGANIZATION | 51 | 0.61 | 1.96 | 0.0016 | 0.0208 |
| DDX3X | GO_REGULATION_OF_T_CELL_MEDIATED_IMMUNITY | 68 | -0.42 | -1.54 | 0.0055 | 0.0276 |
| DDX3X | GO_CYTOKINE_MEDIATED_SIGNALING_PATHWAY | 770 | -0.24 | -1.15 | 0.0345 | 0.0955 |
| DTX1 | GO_CYTOKINE_PRODUCTION | 747 | 0.27 | 1.26 | 0.0052 | 0.0669 |
| DTX1 | GO_REGULATION_OF_SIGNAL_TRANSDUCTION_BY_P53_CLASS_MEDIATOR | 175 | 0.36 | 1.46 | 0.0086 | 0.0858 |
| DUSP2 | GO_CYTOKINE_PRODUCTION | 747 | 0.33 | 1.49 | 0.0011 | 0.0246 |
| DUSP2 | GO_COLLAGEN_FIBRIL_ORGANIZATION | 51 | 0.45 | 1.48 | 0.0267 | 0.1301 |
| **Gene** | **Gene Ontology terms** | **Set size** | **ES** | **NES** | **p value** | **q value** |
| DUSP2 | GO_RESPONSE_TO_INTERFERON_GAMMA | 195 | 0.33 | 1.30 | 0.0315 | 0.1460 |
| DUSP2 | GO_FIBRINOLYSIS | 28 | 0.51 | 1.48 | 0.0337 | 0.1460 |
| EP300 | GO_CHROMOSOME_ORGANIZATION | 1174 | 0.26 | 1.28 | 0.0018 | 0.0032 |
| EP300 | GO_METHYLATION | 356 | 0.31 | 1.41 | 0.0019 | 0.0032 |
| EP300 | GO_RESPONSE_TO_INTERFERON_GAMMA | 195 | -0.50 | -2.15 | 0.0020 | 0.0032 |
| EP300 | GO_REGULATION_OF_T_HELPER_1_TYPE_IMMUNE_RESPONSE | 27 | -0.64 | -1.88 | 0.0020 | 0.0032 |
| EP300 | GO_REGULATION_OF_T_CELL_MEDIATED_IMMUNITY | 68 | -0.59 | -2.17 | 0.0021 | 0.0032 |
| EP300 | GO_CYTOKINE_PRODUCTION | 747 | -0.45 | -2.21 | 0.0022 | 0.0032 |
| EP300 | GO_REGULATION_OF_T_HELPER_17_TYPE_IMMUNE_RESPONSE | 19 | -0.61 | -1.66 | 0.0140 | 0.0124 |
| EZH2 | GO_CHROMATIN_ASSEMBLY_OR_DISASSEMBLY | 180 | -0.40 | -1.63 | 0.0026 | 0.0618 |
| EZH2 | GO_CYTOKINE_MEDIATED_SIGNALING_PATHWAY | 770 | -0.25 | -1.19 | 0.0035 | 0.0618 |
| FAS | GO_CYTOKINE_PRODUCTION | 747 | -0.31 | -1.45 | 0.0020 | 0.0117 |
| FAS | GO_INTERFERON_GAMMA_MEDIATED_SIGNALING_PATHWAY | 89 | 0.41 | 1.50 | 0.0059 | 0.0270 |
| GNA13 | GO_REGULATION_OF_NOTCH_SIGNALING_PATHWAY | 96 | 0.49 | 1.70 | 0.0040 | 0.0857 |
| GNA13 | GO_RESPONSE_TO_INTERFERON_GAMMA | 195 | -0.30 | -1.34 | 0.0105 | 0.1189 |
| IRF4 | GO_REGULATION_OF_T_CELL_MEDIATED_IMMUNITY | 68 | -0.44 | -1.67 | 0.0029 | 0.0188 |
| IRF4 | GO_RESPONSE_TO_INTERFERON_GAMMA | 195 | -0.33 | -1.47 | 0.0045 | 0.0227 |
| IRF4 | GO_GLYCOLYTIC_PROCESS | 104 | 0.39 | 1.38 | 0.0343 | 0.0684 |
| IRF4 | GO_REGULATION_OF_T_HELPER_17_TYPE_IMMUNE_RESPONSE | 19 | -0.54 | -1.54 | 0.0457 | 0.0893 |
| IRF8 | GO_CYTOKINE_PRODUCTION | 747 | -0.44 | -1.99 | 0.0013 | 0.0041 |
| IRF8 | GO_RESPONSE_TO_INTERFERON_GAMMA | 195 | -0.52 | -2.13 | 0.0016 | 0.0041 |
| IRF8 | GO_REGULATION_OF_T_CELL_MEDIATED_IMMUNITY | 68 | -0.45 | -1.54 | 0.0120 | 0.0173 |
| IRF8 | GO_FIBROBLAST_PROLIFERATION | 86 | -0.42 | -1.53 | 0.0146 | 0.0200 |
| IRF8 | GO_FIBROBLAST_ACTIVATION | 10 | -0.69 | -1.56 | 0.0209 | 0.0272 |
| KMT2D | GO_CHROMOSOME_ORGANIZATION | 1174 | 0.31 | 1.59 | 0.0017 | 0.0049 |
| KMT2D | GO_REGULATION_OF_T_CELL_MEDIATED_IMMUNITY | 68 | -0.65 | -2.43 | 0.0020 | 0.0049 |
| KMT2D | GO_REGULATION_OF_T_HELPER_17_TYPE_IMMUNE_RESPONSE | 19 | -0.72 | -2.05 | 0.0020 | 0.0049 |
| KMT2D | GO_RESPONSE_TO_INTERFERON_GAMMA | 195 | -0.39 | -1.75 | 0.0020 | 0.0049 |
| KMT2D | GO_CYTOKINE_PRODUCTION | 747 | -0.37 | -1.88 | 0.0023 | 0.0049 |
| KMT2D | GO_REGULATION_OF_T_HELPER_1_TYPE_IMMUNE_RESPONSE | 27 | -0.60 | -1.89 | 0.0041 | 0.0077 |
| KMT2D | GO_METHYLATION | 356 | 0.32 | 1.49 | 0.0053 | 0.0093 |
| MPEG1 | GO_GLUCOSE_METABOLIC_PROCESS | 117 | 0.41 | 1.50 | 0.0068 | 0.0633 |
| MPEG1 | GO_CYTOKINE_MEDIATED_SIGNALING_PATHWAY | 770 | -0.28 | -1.37 | 0.0120 | 0.0793 |
| MYD88 | GO_REGULATION_OF_NIK_NF_KAPPAB_SIGNALING | 106 | -0.42 | -1.68 | 0.0025 | 0.0309 |
| MYD88 | GO_CYTOKINE_PRODUCTION | 747 | -0.24 | -1.19 | 0.0145 | 0.0967 |
| NOTCH1 | GO_REGULATION_OF_T_CELL_MEDIATED_IMMUNITY | 68 | 0.43 | 1.52 | 0.0105 | 0.0366 |
| NOTCH1 | GO_REGULATION_OF_INNATE_IMMUNE_RESPONSE | 433 | 0.27 | 1.23 | 0.0212 | 0.0612 |
| NOTCH1 | GO_REGULATION_OF_I_KAPPAB_PHOSPHORYLATION | 6 | 0.80 | 1.59 | 0.0233 | 0.0654 |
| NOTCH2 | GO_I_KAPPAB_PHOSPHORYLATION | 18 | 0.61 | 1.67 | 0.0070 | 0.0479 |
| NOTCH2 | GO_CYTOKINE_PRODUCTION | 747 | 0.24 | 1.18 | 0.0181 | 0.0833 |
| NOTCH2 | GO_REGULATION_OF_NOTCH_SIGNALING_PATHWAY | 96 | 0.38 | 1.44 | 0.0183 | 0.0833 |
| **Gene** | **Gene Ontology terms** | **Set size** | **ES** | **NES** | **p value** | **q value** |
| NOTCH2 | GO_ACTIVATION_OF_NF_KAPPAB_INDUCING_KINASE_ACTIVITY | 18 | 0.58 | 1.58 | 0.0233 | 0.0981 |
| PIM1 | GO_REGULATION_OF_T_HELPER_1_TYPE_IMMUNE_RESPONSE | 27 | -0.70 | -2.13 | 0.0023 | 0.0072 |
| PIM1 | GO_REGULATION_OF_T_CELL_MEDIATED_IMMUNITY | 68 | -0.65 | -2.39 | 0.0025 | 0.0072 |
| PIM1 | GO_REGULATION_OF_NIK_NF_KAPPAB_SIGNALING | 106 | -0.44 | -1.74 | 0.0027 | 0.0072 |
| PIM1 | GO_RESPONSE_TO_INTERFERON_GAMMA | 195 | -0.47 | -2.04 | 0.0030 | 0.0072 |
| PIM1 | GO_CYTOKINE_PRODUCTION | 747 | -0.41 | -1.99 | 0.0043 | 0.0072 |
| PIM1 | GO_REGULATION_OF_T_HELPER_17_TYPE_IMMUNE_RESPONSE | 19 | -0.74 | -2.08 | 0.0045 | 0.0072 |
| PRDM1 | GO_MYD88_DEPENDENT_TOLL_LIKE_RECEPTOR_SIGNALING_PATHWAY | 36 | 0.57 | 1.69 | 0.0032 | 0.0260 |
| PRDM1 | GO_REGULATION_OF_B_CELL_RECEPTOR_SIGNALING_PATHWAY | 27 | 0.63 | 1.76 | 0.0033 | 0.0260 |
| SGK1 | GO_REGULATION_OF_T_CELL_MEDIATED_IMMUNITY | 68 | -0.51 | -1.86 | 0.0028 | 0.0216 |
| SGK1 | GO_RESPONSE_TO_INTERFERON_GAMMA | 195 | -0.48 | -2.05 | 0.0034 | 0.0216 |
| SGK1 | GO_CYTOKINE_PRODUCTION | 747 | -0.34 | -1.66 | 0.0064 | 0.0216 |
| SGK1 | GO_COLLAGEN_FIBRIL_ORGANIZATION | 51 | 0.50 | 1.60 | 0.0112 | 0.0266 |
| SOCS1 | GO_CYTOKINE_PRODUCTION | 747 | 0.38 | 1.62 | 0.0013 | 0.0194 |
| SOCS1 | GO_RESPONSE_TO_INTERFERON_GAMMA | 195 | 0.49 | 1.85 | 0.0016 | 0.0194 |
| STAT3 | GO_COLLAGEN_FIBRIL_ORGANIZATION | 51 | 0.65 | 2.23 | 0.0020 | 0.0583 |
| STAT3 | GO_REGULATION_OF_T_CELL_MEDIATED_IMMUNITY | 68 | -0.41 | -1.48 | 0.0184 | 0.1075 |
| STAT6 | GO_CYTOKINE_PRODUCTION | 747 | 0.48 | 1.95 | 0.0010 | 0.0053 |
| STAT6 | GO_RESPONSE_TO_INTERFERON_GAMMA | 195 | 0.57 | 2.09 | 0.0012 | 0.0053 |
| STAT6 | GO_REGULATION_OF_T_HELPER_1_TYPE_IMMUNE_RESPONSE | 27 | 0.64 | 1.73 | 0.0048 | 0.0118 |
| TBL1XR1 | GO_REGULATION_OF_T_CELL_MEDIATED_IMMUNITY | 68 | -0.50 | -1.87 | 0.0027 | 0.0146 |
| TBL1XR1 | GO_RESPONSE_TO_INTERFERON_GAMMA | 195 | -0.35 | -1.53 | 0.0031 | 0.0146 |
| TBL1XR1 | GO_CYTOKINE_PRODUCTION | 747 | -0.29 | -1.46 | 0.0056 | 0.0183 |
| TBL1XR1 | GO_I_KAPPAB_KINASE_NF_KAPPAB_SIGNALING | 261 | -0.29 | -1.30 | 0.0142 | 0.0282 |
| TBL1XR1 | GO_REGULATION_OF_T_HELPER_1_TYPE_IMMUNE_RESPONSE | 27 | -0.51 | -1.56 | 0.0291 | 0.0492 |
| TET2 | GO_CYTOKINE_MEDIATED_SIGNALING_PATHWAY | 770 | 0.33 | 1.46 | 0.0017 | 0.0895 |
| TET2 | GO_T_CELL_MEDIATED_IMMUNITY | 104 | 0.41 | 1.55 | 0.0056 | 0.1798 |
| TNFAIP3 | GO_CYTOKINE_PRODUCTION | 747 | 0.44 | 1.80 | 0.0010 | 0.0068 |
| TNFAIP3 | GO_REGULATION_OF_SIGNAL_TRANSDUCTION_BY_P53_CLASS_MEDIATOR | 175 | 0.42 | 1.55 | 0.0025 | 0.0104 |
| TNFAIP3 | GO_I_KAPPAB_KINASE_NF_KAPPAB_SIGNALING | 261 | 0.37 | 1.40 | 0.0058 | 0.0180 |
| TNFAIP3 | GO_REGULATION_OF_NOTCH_SIGNALING_PATHWAY | 96 | 0.43 | 1.46 | 0.0137 | 0.0278 |
| TNFRSF14 | GO_REGULATION_OF_T_HELPER_17_TYPE_IMMUNE_RESPONSE | 19 | 0.65 | 1.78 | 0.0017 | 0.0860 |
| TP53 | GO_LIPID_METABOLIC_PROCESS | 1219 | 0.34 | 1.54 | 0.0012 | 0.0065 |
| TP53 | GO_REGULATION_OF_T_CELL_MEDIATED_CYTOTOXICITY | 33 | -0.71 | -2.21 | 0.0023 | 0.0065 |
| TP53 | GO_REGULATION_OF_T_HELPER_1_TYPE_IMMUNE_RESPONSE | 27 | -0.65 | -1.93 | 0.0024 | 0.0065 |
| TP53 | GO_REGULATION_OF_NATURAL_KILLER_CELL_MEDIATED_IMMUNITY | 47 | -0.67 | -2.23 | 0.0026 | 0.0065 |
| TP53 | GO_RESPONSE_TO_INTERFERON_GAMMA | 195 | -0.40 | -1.66 | 0.0064 | 0.0093 |
| TP53 | GO_REGULATION_OF_GLUCOSE_METABOLIC_PROCESS | 73 | 0.42 | 1.44 | 0.0257 | 0.0292 |
| ZFP36L1 | GO_CYTOKINE_PRODUCTION | 747 | -0.36 | -1.50 | 0.0010 | 0.0171 |
| ZFP36L1 | GO_RESPONSE_TO_INTERFERON_GAMMA | 195 | -0.38 | -1.43 | 0.0071 | 0.0408 |

ES, enrichment score; NES, normalized enrichment score.
